# Stability of mechanically exfoliated layered monochalcogenides under ambient conditions

**DOI:** 10.1038/s41598-023-46092-1

**Published:** 2023-11-04

**Authors:** Daria Hlushchenko, Anna Siudzinska, Joanna Cybinska, Malgorzata Guzik, Alicja Bachmatiuk, Robert Kudrawiec

**Affiliations:** 1 Lukasiewicz Research Network, PORT Polish Center for Technology Development, Stablowicka 147, 54-066 Wroclaw, Poland; 2 Department of Semiconductor Materials Engineering, Faculty of Fundamental Problems of Science and Technology, Wroclaw University of Science and Technology, Wybrzeze Wyspianskiego 27, 50-370 Wroclaw, Poland; 3 Faculty of Chemistry, University of Wroclaw, F. Joliot-Curie 14, 50-383 Wroclaw, Poland

**Keywords:** Materials for devices, Materials for energy and catalysis, Nanoscale materials

## Abstract

Monochalcogenides of groups III (GaS, GaSe) and VI (GeS, GeSe, SnS, and SnSe) are materials with interesting thickness-dependent characteristics, which have been applied in many areas. However, the stability of layered monochalcogenides (LMs) is a real problem in semiconductor devices that contain these materials. Therefore, it is an important issue that needs to be explored. This article presents a comprehensive study of the degradation mechanism in mechanically exfoliated monochalcogenides in ambient conditions using Raman and photoluminescence spectroscopy supported by structural methods. A higher stability (up to three weeks) was observed for GaS. The most reactive were Se-containing monochalcogenides. Surface protrusions appeared after the ambient exposure of GeSe was detected by scanning electron microscopy. In addition, the degradation of GeS and GeSe flakes was observed in the operando experiment in transmission electron microscopy. Additionally, the amorphization of the material progressed from the flake edges. The reported results and conclusions on the degradation of LMs are useful to understand surface oxidation, air stability, and to fabricate stable devices with monochalcogenides. The results indicate that LMs are more challenging for exfoliation and optical studies than transition metal dichalcogenides such as MoS_2_, MoSe_2_, WS_2_, or WSe_2_.

## Introduction

Layered monochalcogenides (LMs) are among the potential materials for fabricating novel low-dimensional semiconductor devices. They have potential applications in photovoltaic^1–11^, thermoelectric and energy storage devices^12–17^, transistors^18,19^, photodetectors^20–22^, devices utilizing piezoelectricity^12,23,24^, water splitting^25–27^, ferroelectricity^28–30^, optoelectronics^31–35^, memory devices^36^, spintronics^37^, nanotubes and nanowires^38–40^.

There are several theoretical articles related to the stability of LMs and they have focused on materials from group III and IV LMs^41–94^. These studies provide important information on the adsorption of oxide molecules and the mechanism of oxidation, as well as changes in the crystal structure and characteristics of reaction products during oxidation. Guo et al. reported that group III LMs monolayers are considerably sensitive to ambient oxygen. However, an ideal monolayer has a higher oxidation resistance^41^. During the mechanical exfoliation of LMs, which is used for fabricating thin flakes, point defects (such as vacancies and interstitials), dislocations, and grain boundaries appears. Therefore, such monolayers become less resistant to oxidation^41^. In addition, oxidation under ambient conditions can also be related to the exothermic process detected in GeSe^42^. In GeSe, oxidation occurs by breaking Ge-Se bonds and bonding germanium with oxygen^42–45^. Theoretically, SnS and SnSe can be more oxidation resistant because of higher oxidation energy barriers (1.56 and 0.97 eV), which are larger than those of GeS (1.67 eV), GeSe (1.13 eV), and BPs (0.70 eV)^43^. On the other side, materials from group IV LMs are less prone to oxidation than black phosphorus (BPs), but in aqueous environment, these materials degrade in nanoseconds^47^.

The oxidation mechanism was studied experimentally for GeS^50,62,63,74–76^, GeSe^50^ SnS^50,51,62^ , and SnSe^50,53–55,78,79^ from group IV-VI and for GaS^52,94^ and GaSe^52,56–59^ from group III LMs. By using Raman spectroscopy founded that GaSe monolayers can withstand up to 6 h in air before being degraded completely while GaS monolayers proved to be more stable^52^. Further, it is observed that the encapsulation of GaSe and GaS monolayers by polymer films helps avoid degradation at a high laser illumination^52^. Moreover, PMMA encapsulation can increase stability up to six weeks^51^. Humidity strongly influences oxidation mechanisms in most exfoliated LMs, which leads to the formation of oxides such as SeO_2_ and Ga_2_O_3_ in the GaSe material^57^. This conclusion was confirmed by Kowalski et al., who found Raman peaks typical for the aqueous solution of selenic acid during the oxidation of GaSe material^57^. Beechem et al. concluded that the top layers of the oxidized GaSe flake comprises an ultrathin GaSe capped with the combination of Ga_2_Se_3_, α-Se, and Ga_2_O_3_^59^. Furthermore, the appearance of oxide SnO_x_ on the top of SnS^51^ and SnSe^53–55^ has been detected.

These reports indicate that the oxidation of LMs is an important phenomenon that cannot be overlooked in the further use of these materials. However, it is rather difficult to conclude from previous studies which type of LMs are more sensitive or stable because they are not tested under the same conditions and compared with each other. Fabrication strategies, which minimize air exposure or work primarily in an inert atmosphere (e.g., nitrogen or argon gloveboxes), may restrict material degradation^52,60,61^. Controlled oxidation can also be an option to fine-tune material properties and use it in real applications^41^. Therefore, better understanding of the sensitivity of two-dimensional materials to oxygen and water molecules after the exposure to air provides a new perspective on the fabrication of optoelectronic devices with the desired properties.

**Figure 1:**
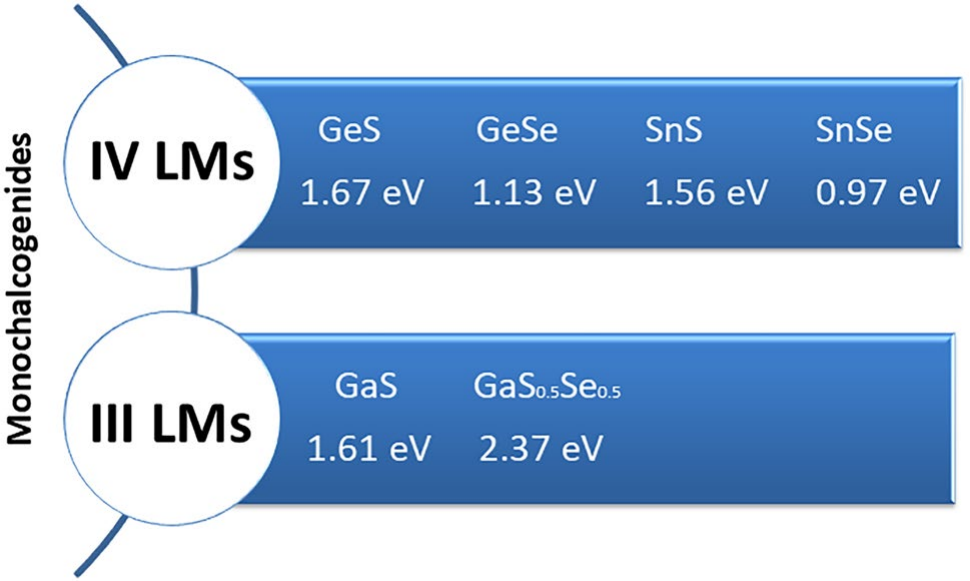
Monochalcogenides studied in this work. Bandgap values for each material are presented for bulk crystals.

In this article, we report a systematic study of mechanically exfoliated flakes of LMs in air conducted over a month from the exfoliation to understand main differences between each material in terms of sensitivity upon ambient exposure. The materials studied carefully and compared in this article are grouped in Fig. 1.

## Experimental section

### Characterization of exfoliated samples

Optical images were estimated with a Leica optical microscope using a 100 × objective lens. The morphologies of the sample surfaces were investigated using an FEI Helios 660 scanning electron microscope (SEM) in a high vacuum. The thickness of thin and thick exfoliated flakes was measured by a Bruker surface profilometer with 100 nm stylus. Monolayer thickness was evaluated immediately after exfoliation by using an optical microscope Keyence VHX-7000N. Energy-dispersive X-ray spectroscopy (EDS) were used for the composition characterization of oxidized flakes. Transmission electron microscopy (TEM), high-resolution TEM (HRTEM), and electron energy loss spectroscopy (EELS) measurements were performed using double-aberration corrected FEI Titan3 60–300 (S)TEM microscope equipped with a high brightness X-FEG. The crystals were mechanically exfoliated onto carbon lacey grid and subsequently imagined, operating at an accelerating voltage of 300 kV, with spherical aberration correction. EELS analysis was performed in the STEM mode using a Gatan Continuum (model 1077) EELS spectrometer with an operating voltage, beam current, EELS aperture size, and dispersion of 300 kV, 120 pA, 5 mm, and 0.3 eV/ch, respectively.

### Raman and photoluminescence measurement

Confocal micro-Raman spectrometer (WiTEC) Alpha 300 R with laser of a 532 nm wavelength was used to measure Raman and photoluminescence (PL) spectra. Our spectrometer allows for the selecting two diffraction gratings: 1200 g/cm and 300 g/cm. The Raman and PL spectra were measured with grating 1200 g/cm and 300 g/cm respectively. The Raman signal was collected in a backscattering configuration through a 100 × objective with numerical aperture 0.95 and spatial resolution down to ~ 200 nm. The spectra were taken with the same accumulation time. Measurements were conducted in ambient air at laboratory conditions. To ensure the repeatability of measurements, signal intensity was calibrated according to the silicon (Si) peak before recording each spectrum. For measurements, a laser power was selected to minimize the heating and photodegradation of studied materials and receive the optimal signal to noise ratio. The conducted experiment on the selection of laser power for each material was placed in the electronic supplementary materials (ESM) (Figs. S1–S7). From experiment, the lowest laser power (0.05 mW) has been applied for all materials.

## Results

**Figure 2:**
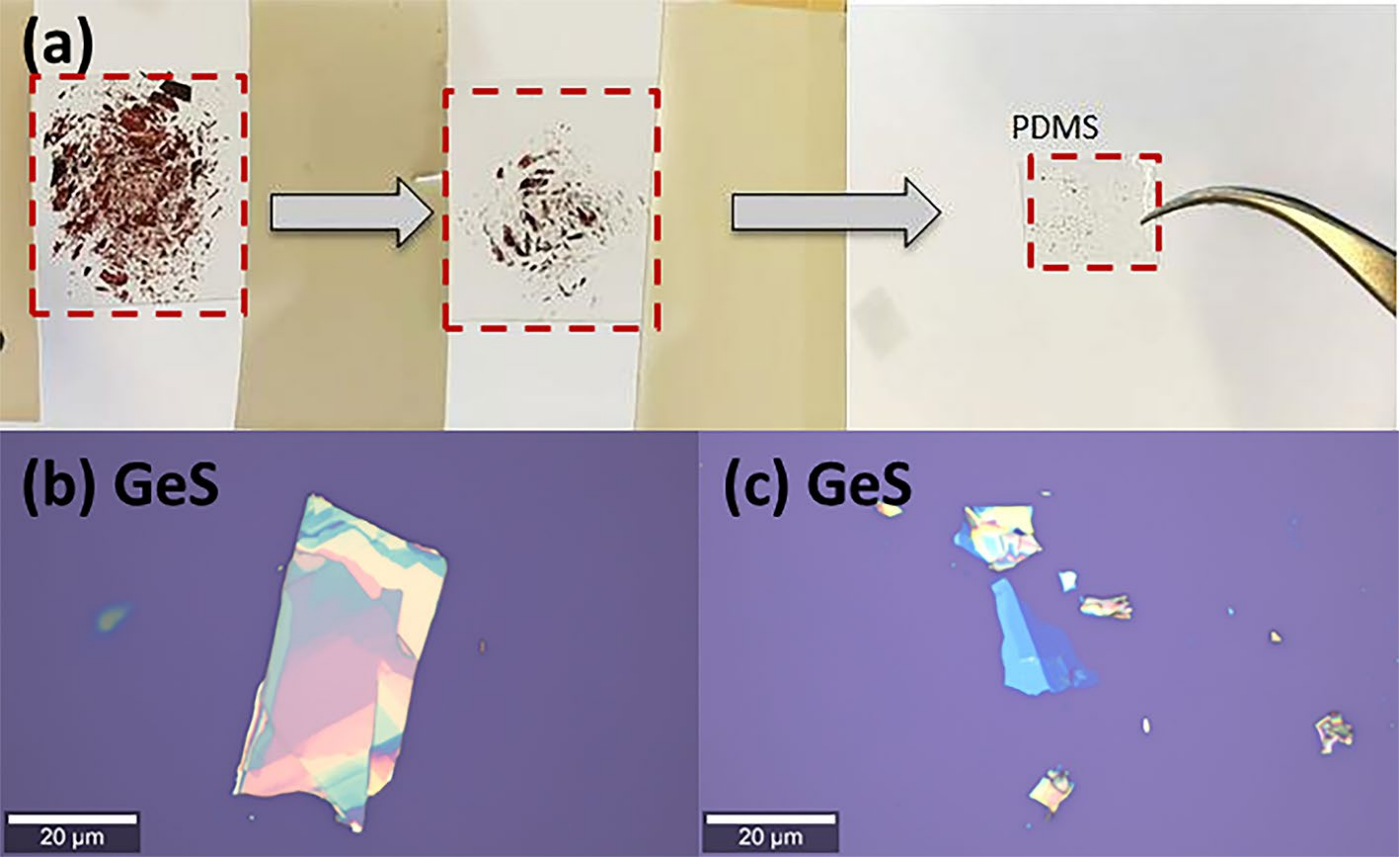
(**a**) Mechanical exfoliation process of GeS crystal. GeS thick flakes are thinned and transferred into polydimethylsiloxane (PDMS); (**b**) Image of freshly exfoliated thick GeS flake; (**c**) Image of freshly exfoliated GeS few-layer.

Mechanical exfoliation is the most straightforward approach to create atomically thin flakes, especially in the early stages of material characterization and semiconductor device fabrication, *i.e.,* device prototyping. The LMs show strong covalent bonding in the two-dimensional plane and a strong interlayer force attributed to the lone pair electrons, which generate a large electron distribution and electronic coupling between adjacent layers^60,61^. Owing to antiferroelectric coupling between the in-plane polarized few-layers, it is difficult to receive single monolayers from bulk monochalcogenide crystal^41,62^. In comparison to transition metal dichalcogenides, monochalcogenides LMs are more difficult to exfoliate, and therefore, studies on exfoliating such materials and those that compare exfoliation difficulties in this family of layered materials remain limited^60–65^. The thin and thick flakes are obtained by mechanical exfoliation using the Scotch-tape technique (Fig. 2), the physical mechanism of which was described by Gao et al. ^60^. The thickness of such flakes decreases with - geometric progress^64^. All studied materials were synthetic and purchased from HQ graphene and the 2D Semiconductors company. Images of freshly exfoliated GaS, GaS_0.5_Se_0.5_, GeS, SnSe monolayers are presented in Fig. S8 in the ESM.

To study the degradation mechanism in LMs, flakes were exfoliated directly on Si with a SiO_2_ oxide thickness of 300 nm. The flakes were exfoliated in the glovebox with a nitrogen atmosphere, where O_2_ and H_2_O < 1 parts per million (ppm) were ensured. After exfoliation, the flakes are left exposed to the open laboratory air for variable time intervals from the start of exfoliation till the end of the month. For freshly exfoliated flakes, Raman and PL spectra were immediately measured and compared with the air-exposed ones after they were removed from the glovebox.

To detect the stability of mechanically exfoliated LMs, we use Raman spectroscopy because of the high sensitivity for monitoring the oxidation process by analyzing Raman modes (e.g., A_g_(1) and E_g_(2)). First, Raman spectroscopy allows us to observe new Raman modes on oxidized flakes. Spectral positions of new Raman peaks provide information about the type of oxides. Further, the intensity of Raman peaks related to LM should decrease with material degradation via oxidation. Given the above-mentioned reasons, Raman spectroscopy is the first marker we used to study the degradation/oxidation process in LMs. The second marker is the photoluminescence intensity because in the first approximation, this signal is proportional to the volume of the material that is excited. In addition, PL intensity should decrease with material degradation because of stronger non-radiative recombination. These two types of markers (*i.e.*, the intensity of Raman peaks and PL signal) are not quantitatively accurate. However, it is difficult to find better ones that are readily available and noninvasive. For each material the oxidation study was performed for flakes from monolayers to thicker ones. Each flake was carefully measured and specific oxidation trend is presented.

During the exfoliation of LMs, a strong dependence of the lateral crystal size on the thickness of LMs flakes was noticed: reducing the thickness makes the lateral size smaller^64^. This is a well-known phenomenon and the typical flake sizes are listed in Table 1. These sizes vary depending on the material and they are slightly larger for group III LMs materials. Using mechanical exfoliation, we obtained the monolayers for GeS, SnS, GaS, GaS_0.5_Se_0.5_. However, for GeSe and SnSe, mechanical exfoliation is more difficult. This is in accordance with a previous study where authors attributed this difficulty to possible antiferroelectric coupling between layers^64,68,69^. Jiang et al. synthesized single-layer rectangular SnSe flakes by a vapor transport deposition method, followed by a nitrogen etching technique^69^. This suggests that the difficulties of mechanical exfoliation are not an obstacle to the direct synthesis of LMs with monolayer thickness. To summarize the difficulty of exfoliation, all tested materials were divided into groups based on the hardening of exfoliation (Table 1). In conclusion, we observe the easier exfoliation of sulfur-containing materials where monolayer is obtainable.

**Table 1:** Difficulty with the mechanical exfoliation of group IV and III LMs. Low hardening – materials hard to exfoliate and receive monolayer; Strong hardening – easy to exfoliate, monolayer is obtainable.

Material	Layer size (µm)	Lowest thickness (ML)	Hardening of exfoliation		
			Low	Medium	Strong
**GeS**	~ 10 × 5	~ 1–2		X	
**GeSe**	~ 25 × 15	~ 15–20	X		
**SnS**	~ 10 × 5	~ 1–2		X	
**SnSe**	~ 20 × 15	~ 30–35	X		
**GaS**	~ 30 × 20	~ 1–2			X
**GaS**_**0.5**_**Se**_**0.5**_	~ 60 × 40	~ 1–3			X

### Surface degradation of group IV layered monochalcogenides

**Figure 3:**
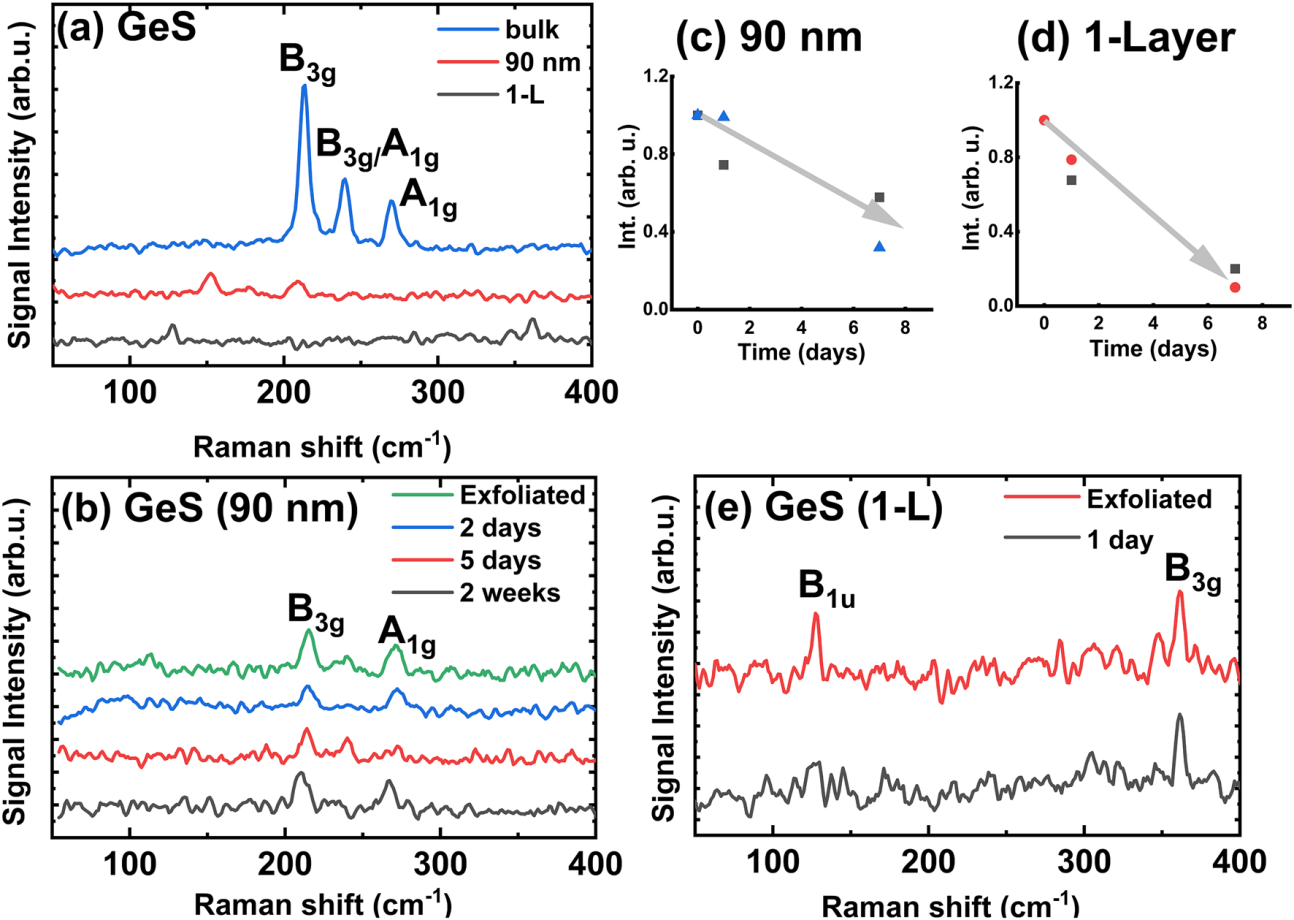
Raman spectra for: (**a**) Bulk GeS crystal and exfoliated 90 nm and 1-L thick flake; (**b**) GeS flake with a thickness of 90 nm: freshly exfoliated and from one week to two weeks of exposure in an ambient environment; (**c**) Raman peaks intensity vs oxidation time for GeS 90 nm flake; (**d**) Raman peaks intensity vs oxidation time for GeS 1-L flake; (**e**) Freshly exfoliated GeS 1-L flake after one day of exposure in an ambient environment.

GeS and GeSe are layered orthorhombic crystals with band gaps of 1.67 and 1.13 eV, respectively^43–45,74^. Figure 3 shows the Raman spectra for the exfoliated and air-exposed GeS flakes. For the bulk material, three intense peaks corresponding to the B_3g_, B_3g_/A_1g_, and A_1g_ modes are observed (Fig. 3a). The positions of these modes are in agreement with those previously reported in Ref^62,63^. We observed that the intensities of the Raman peaks does not change significantly with increasing oxidation time, indicating that the oxidation of the upper layer is caused by direct contact with the environment (Fig. 3b–c). In this case, upper layer can prevent the oxidation of subsequent layers underneath.

For GeS monolayer a few Raman modes were detected (B_1u_ and B_3g_) at positions 127 and 361 cm^-1^ respectively (Fig. 3e). Park et al*.* was calculated and presented Raman shifts for four GeS peaks (B_1u_, B_2g_, A_g_(2)^2^, B_3g_(2))^2^, where Raman shifts increase or decrease depending on the number of layers^46^. A decrease in the intensity can also be recognized for monolayer flake (see Fig. 3d–e). However, the intensity of the Raman peak is very weak right after exfoliation. In addition, the Raman peaks for monolayer GeS disappear during measurements, indicating their low stability under ambient conditions or/and illumination by laser light (Fig. 3e).

**Figure 4:**
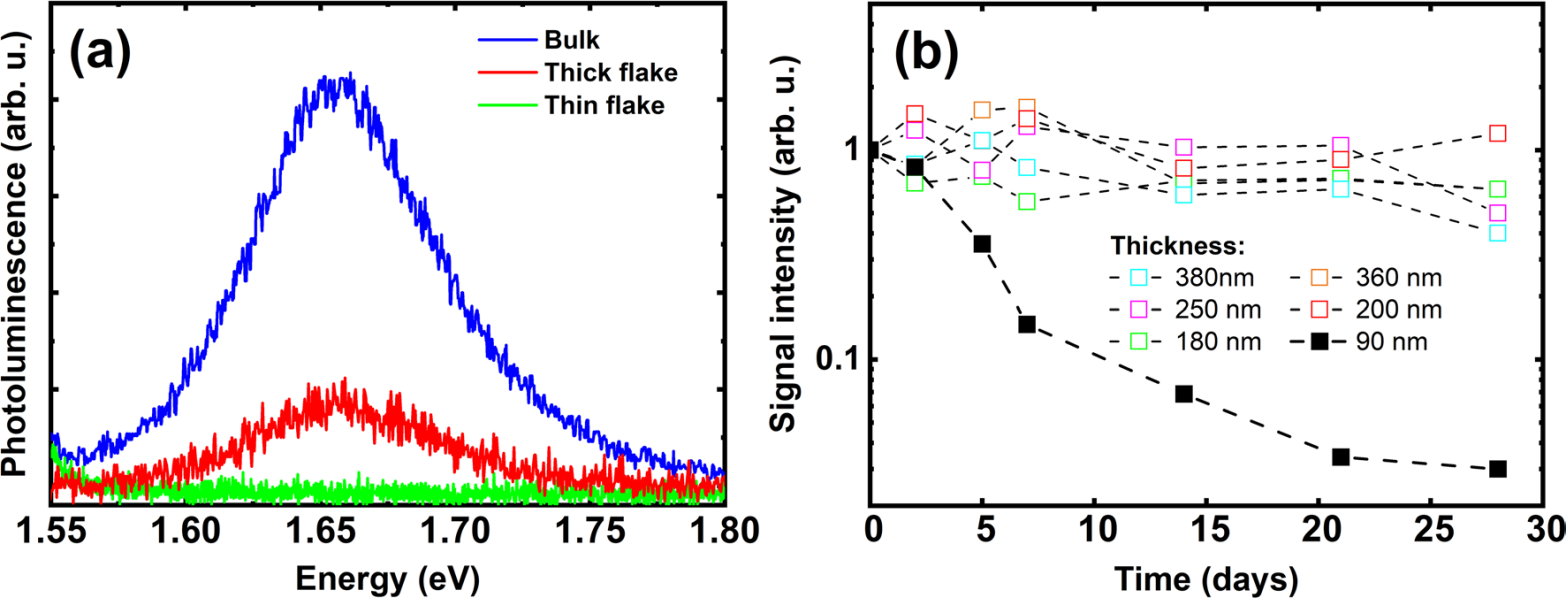
(**a**) Photoluminescence comparison spectra for freshly exfoliated bulk, few-layer, and thick GeS flakes; (**b**) Photoluminescence stability for GeS few-layer, thin, and thick flakes during exposure to ambient air, normalized at the beginning.

The PL for bulk GeS is visible at 1.68 eV, whereas for few-layer GeS no PL is detected at room temperature at this excitation power (0.05 mW), see Fig. 4a. The PL is on the same level when thick flakes (> 200 nm) are exposed to air, as shown in Fig. 4b. For the thinner flake (thickness = 90 nm), PL decreased monotonically with time. These differences can be explained by the saturation of the thickness of the oxidized layer on the top of GeS.

Since PL comes from the volume of the material, the oxidized layer of the same thickness is more important in thin flakes than in thick ones. Therefore, for thin GeS flakes, the PL decreases with time because it degrades due to oxidation. Thus, the observation of PL from atomic-thick GeS layers remains very challenging. These types of layers require rapid protection immediately after exfoliation or storage in a suitable atmosphere.

**Figure 5:**
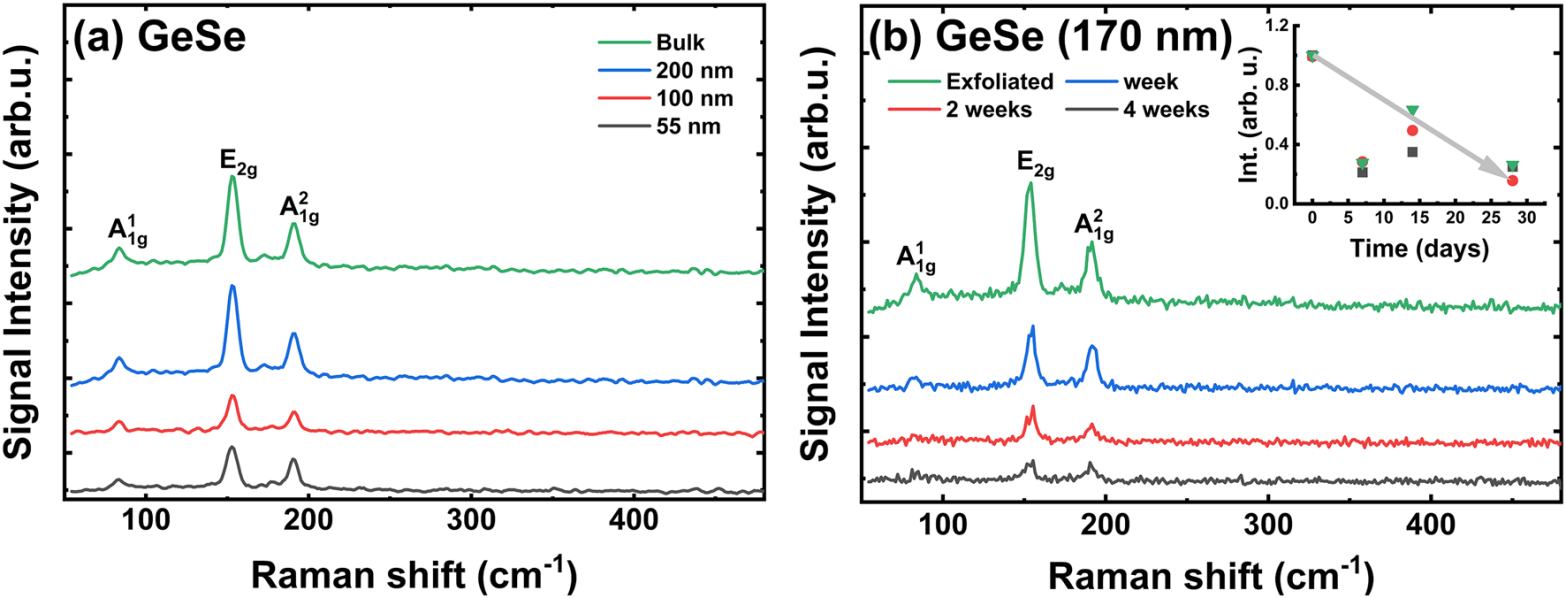
Raman spectra for: (**a**) Exfoliated GeSe flakes with different thickness after exfoliation; (**b**) GeSe flake with a thickness of 170 nm for different periods of exposition under laboratory conditions.

For understanding the main differences between the stability of GeS and GeSe materials, we repeated all measurements for the exfoliated GeSe. The bulk GeSe crystal is a semiconductor with an indirect band gap at ~ 1.13 eV^43–45,49,62,71,75,78^ and therefore, no PL is observed for the GeSe flakes. The Raman spectra for freshly exfoliated and air-exposed GeSe flakes are shown in Fig. 5. Given the difficulty in carrying out the exfoliation for this material, the thin layers were excluded. For exfoliated thick flakes, the A_1g_(1), A_1g_(2), E_1g_, and E_2g_ modes identified in the Raman spectra are consistent with those reported in Refs.^71,75^, see Fig. 5a. The first measured Raman spectra, immediately after exfoliation, was chosen as the intensity reference. The material stability study shows a decrease the intensities of Raman peaks, where the lowest ones was detected after 4 weeks of oxidation in laboratory conditions (Fig. 5b).

The comparison of the degradation effects in GeS and GeSe materials indicates higher oxidation for the GeSe material. The main reason is the presence of Se in GeSe, and the Se protrusions during exposure at ambient conditions. The results from EDS confirm the oxidation process in this material (see Figs. S9–S12 in ESM).

**Figure 6:**
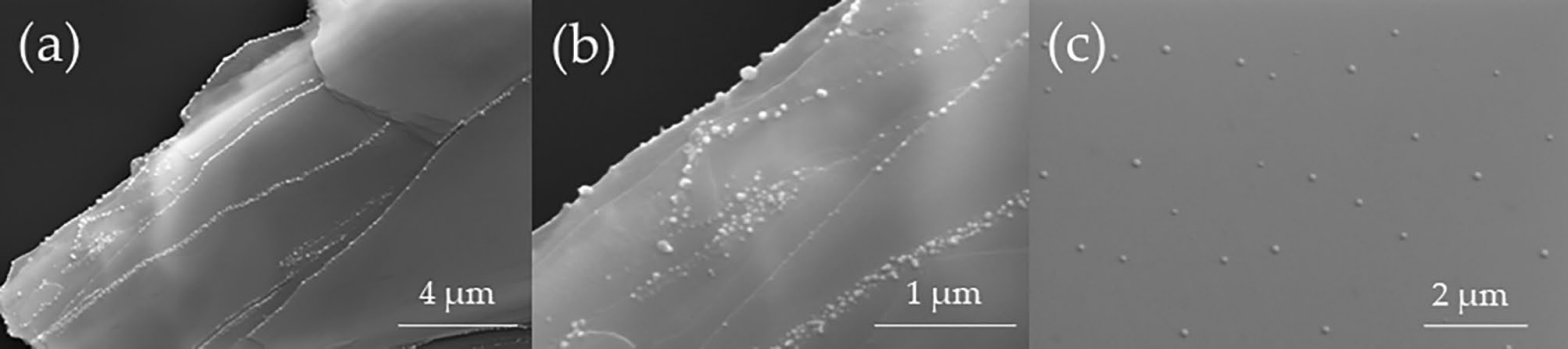
SEM images of GeSe thick flake exfoliated on the SiO_2_/Si substrate: (**a**) View on side edge of thin GeSe flake; (**b**) Zoom on protrusions on side edge of thin GeSe flake; (**c**) Protrusions in the middle of the thin GeSe flake. Images were taken for exfoliated GeSe thin flake after one month exposure under laboratory conditions.

As shown in Fig. 6, the characterization of the oxidized flakes by SEM indicates two types of protrusions. The first type are on the edges of each layer flake (Fig. 6a), whereas the other ones are observed on the entire surface of the thick flakes (Fig. 6b). The smaller protrusions were noticed in the middle of oxidised flake (Fig. 6c), which may also indicate oxidation of the flake in the center of the sample. A similar type of protrusion is found for the layered HfSe_2_^77^. In HfSe_2_, they are related by the growth of Se-rich blisters when Hf oxidizes into HfO_2_ and eliminates Se at the flake surface. The mechanism is similar during GeSe exfoliation under laboratory conditions. The protrusions at flake step-edges have a higher density because of dangling bonds that appear at these edges after mechanical exfoliation. These dangling bonds can act as the most appropriate nucleation sites for the growth of protrusions^77^. For protrusions on step-edges of the flake, EDX maps were collected and presented at ESM (Fig. S13 in ESM). The collected maps confirm that the protrusions contain selenium (Se). Such features were not observed for GeS flakes, which means that the observed phenomenon is related to the Se component of the material that is oxidized; thus, in the case of monochalcogenides and dichalcogenides, it is GeSe and HfSe_2_, respectively.

**Figure 7:**
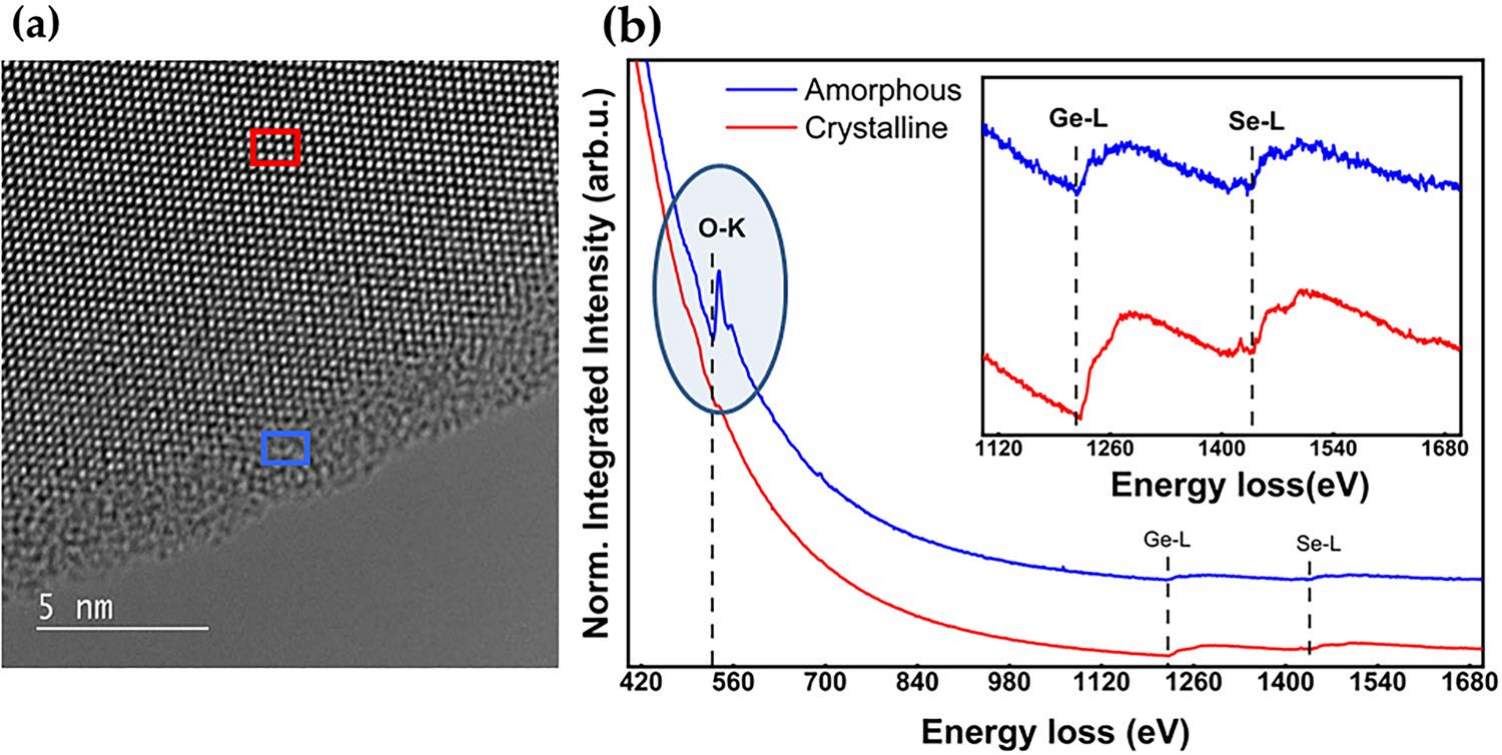
(**a**) STEM image of the GeSe flake. The areas marked by the red and blue rectangles indicate the crystalline and amorphous phases of the material, respectively; (**b**) EELS spectra for the amorphous and crystalline phases.

**Figure 8:**
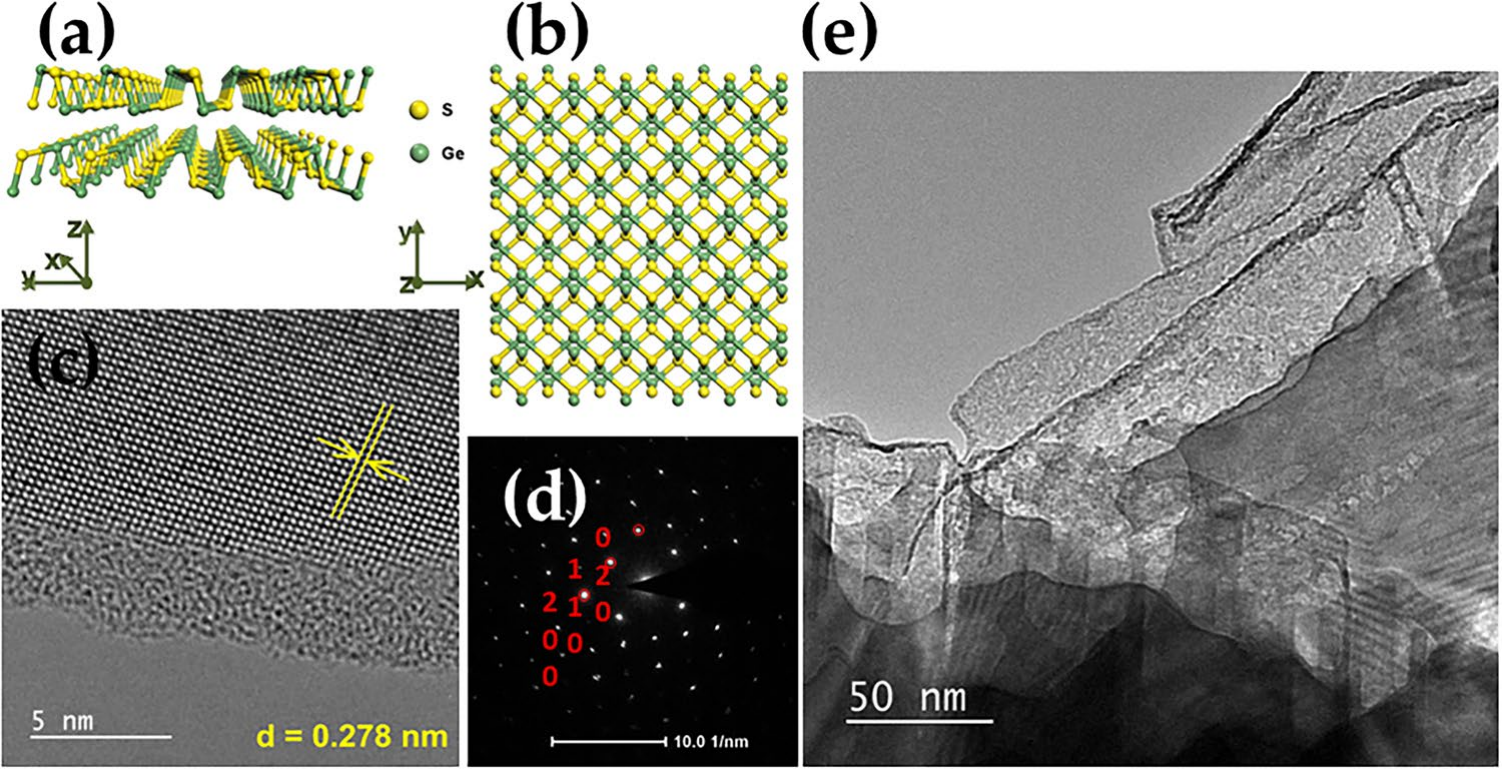
Top (**a**) and side (**b**) view of the crystal structure for single layer GeS; ((**c**) and (**d**)) HRTEM image and corresponding FFT image of the few-layer GeS flake; ((**c**) and (**d**)) TEM analysis of the few-layer GeS flake; (**e**) HRTEM image of the few-layer GeS flake surface.

**Figure 9:**
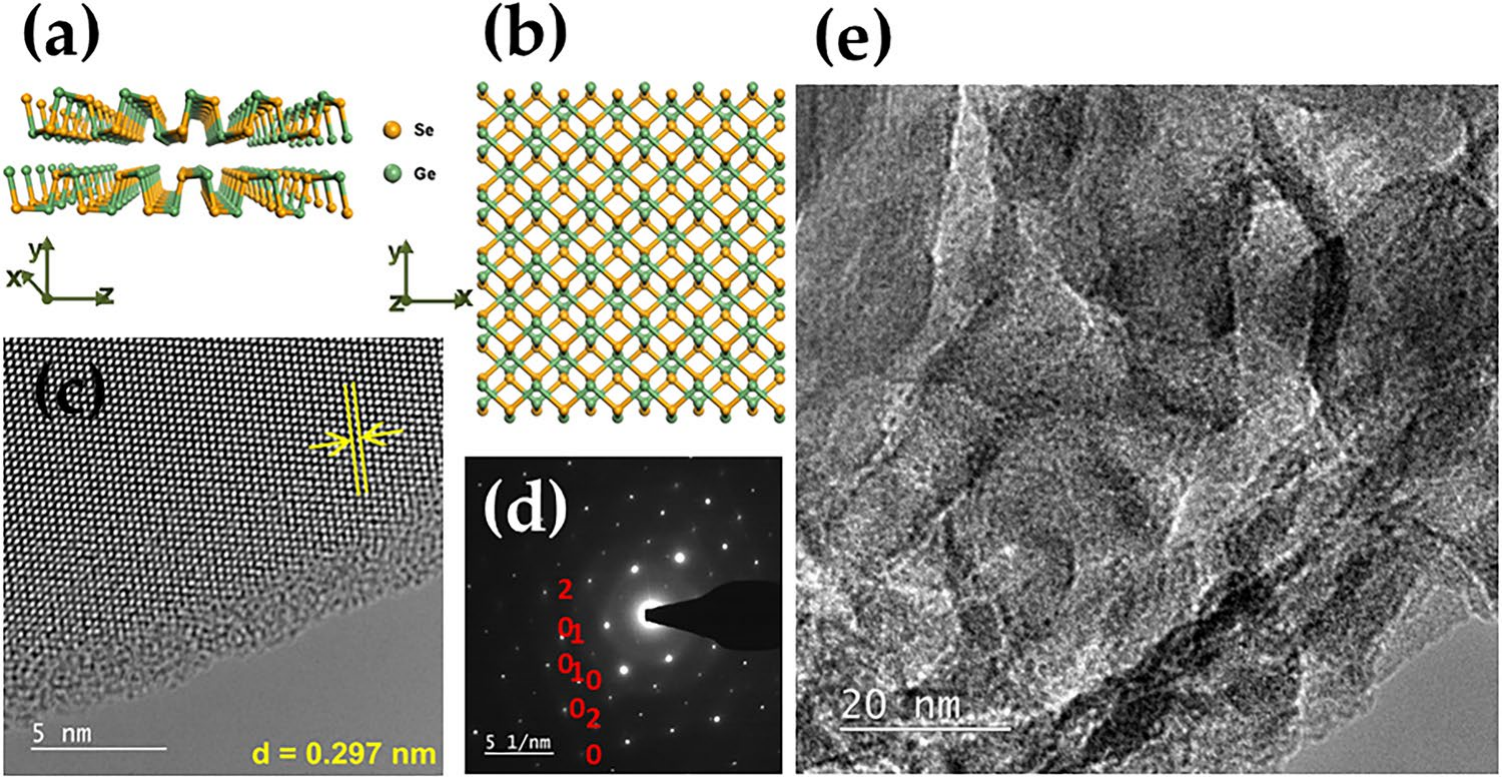
Top (**a**) and side (**b**) view of the crystal structure for single layer GeSe; ((**c**) and (**d**)) HRTEM image and corresponding FFT image of the few-layer GeSe flake; ((**c**) and (**d**)) TEM analysis of the few-layer GeSe flake; (**e**) HRTEM image of the few-layer GeSe flake surface.

Detailed observations of flake degradation are established for the GeS and GeSe material using STEM and EELS spectroscopy (Figs. 7, 8, 9). The TEM images of the studied flakes are represented in Figs. 8 and 9. The EELS spectra for GeSe are collected from the crystalline (red rectangle in Fig. 7a) and amorphous areas (blue rectangle in Fig. 7a). Figure 7b shows that the oxygen (O–K) content is clearly visible for the amorphous material. As these measurements are performed under vacuum conditions, the process is attributed to the interaction of the electron beam with the thin layered material. However, some oxidation occurs during the transfer of the sample to the microscope. In this case, beam irradiation may accelerate the oxidation process, what was observed and shown in Anim. 1 and Anim. 2 in the ESM. A very important conclusion from this observation is that the process of flake degradation is anisotropic, *i.e.,* stronger degradation is observed at the edges of the fake. The same can be expected during oxidation in ambient conditions. Ge bounds in GeS and GeSe flakes are saturated on the surface of the flakes while they are more active at flake edges and can be easily saturated by oxygen, leading to stronger flake oxidation from edges. On the flake surface, the oxidation process is promoted by S (Se) vacancies or other defects. The stronger degradation of GeSe flakes can be attributed to the poorer surface quality (higher concentration of Se vacancy), which correlates with the observation of Se protrusions on the surface shown in Fig. 6.

SnS and SnSe are other representatives of group IV LMs. SnS has an indirect band gap of ~ 1.56 eV for bulk^43,45,49,62,71,78,79,90–92^. For the bulk SnSe, the band gap is indirect at ~ 0.97 eV for the monolayer and it decreases with an increase in the number of layers^78–82^.

**Figure 10:**
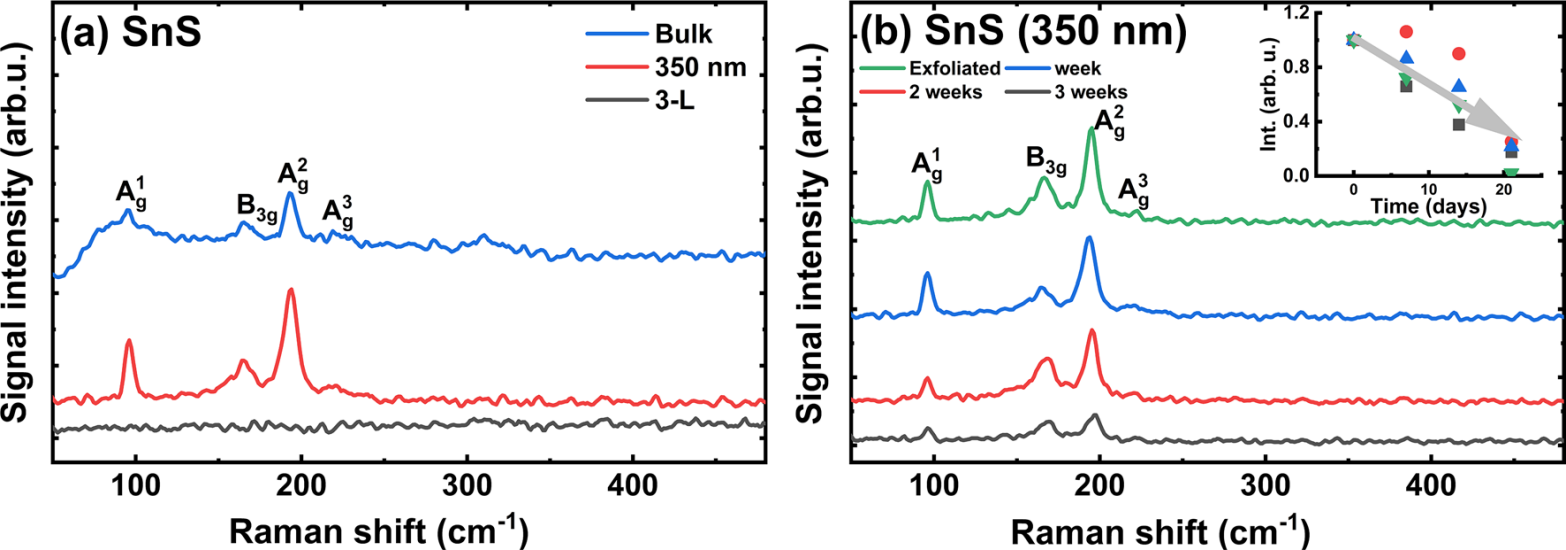
Raman spectra for: (**a**) Bulk, exfoliated thick, and few-layer SnS; (**b**) SnS thick flake after different exposition times under laboratory air.

In the Raman spectra of the SnS samples, five modes are clearly visible (A_g_(1), A_g_(2), A_g_(3), B_2g_, and B_3g_), see Fig. 10a. The positions of SnS modes are previously reported^80,81,83,84^. For bulk SnS we noticed a raised background, which could be related to impurities on the crystal surface.

Figure 10b shows the Raman spectra of a thick SnS flake recorded at different time intervals. In this case, a decrease in the intensity of the Raman modes is clearly observed and attributed to sample degradation with time (Inset in Fig. 10b). This effect is considerably stronger for very thin SnS flakes (few-layers) because the disappearance of Raman modes is observed during measurements. This observation confirms the immediate degradation of the thin SnS flakes after exfoliation when the sample is exposed to air. Similar decrease of signal intensity was observed for other flakes of SnS and SnSe. In addition, the effect of the oxidation of the SnS and SnSe flakes is clearly confirmed by EDS (see Figs. S14–S17 in ESM).

**Figure 11:**
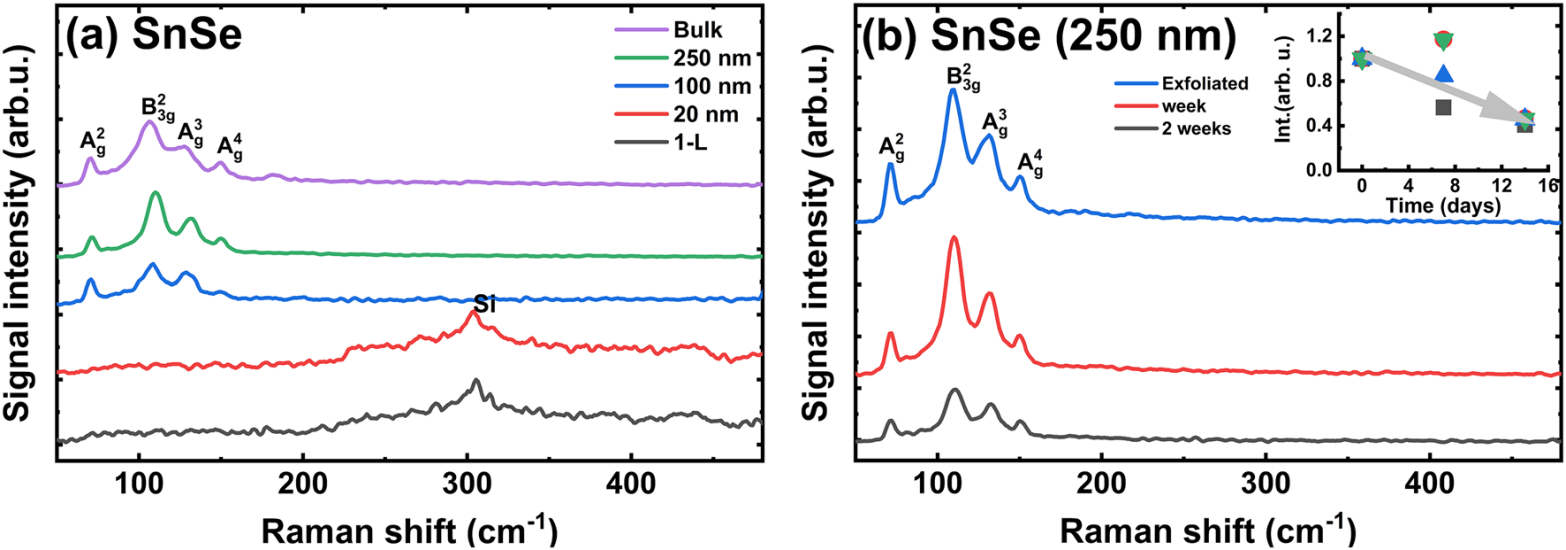
Raman spectra for: (**a**) SnSe of different thicknesses after exfoliation; (**b**) SnSe thick flake after different exposition times under laboratory air.

The Raman spectra for the bulk SnSe shows the A_g_(2), A_g_(3), A_g_(4), and B_2g_(3) modes at positions 71, 112, 133, and 150 cm^−1^, respectively (Fig. 11). These modes were previously characterized by Yang et al.^82^. The thick flakes (up to 100 nm) show these peaks. However, for thin flakes and few-layers only peak from Si is visible (Fig. 11a).

### Surface degradation of group III layered monochalcogenides

**Figure 12:**
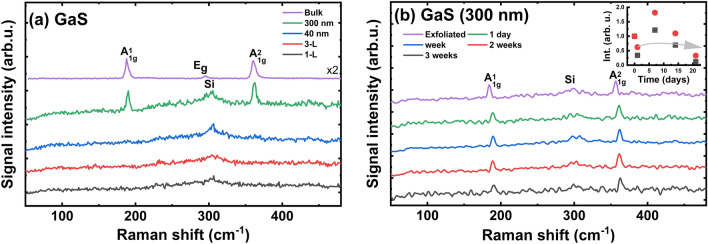
Raman spectra for: (**a**) GaS of different thicknesses after exfoliation; (**b**) GaS thick flake after various exposition times in laboratory conditions.

GaS has an indirect band gap of 1.61 eV in laboratory conditions^52,86,87,94^, and it retains this indirect band gap with thickness reduced up to the single layer^52^. Therefore, band gap-related emission is difficult to observe for these samples and cannot be used as a marker for material degradation study. In this case, Raman measurements were used to evaluate the degradation process of the GaS flakes. Figure 12 shows the Raman spectra for the GaS exfoliated flakes. The intensity of Raman peaks strongly depends on the thickness of the GaS flake (Fig. 12a), which is consistent with the previous studies on this material^86,87^.

Two modes are visible for bulk and thick flakes: A_1g_(1) and A_1g_(2) (Fig. 12). In the case of monolayer, only peak from substrate is visible, see Fig. 12a. The intensities of GaS modes after a few days of exposure changed slightly. A decrease in the intensity was observed after three weeks, which indicates the considerably high stability of GaS under ambient conditions. This statement is similar with the previously reported by Afaneh et al.^52^. The appearance of oxygen on the surface is confirmed with EDS analysis as shown in Figs. S18–S19 in ESM.

**Figure 13:**
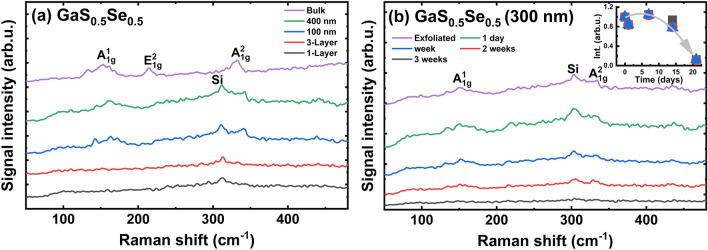
Raman spectra for: (**a**) GaS_0.5_Se_0.5_ bulk and freshly exfoliated flakes; (**b**) Raman spectra for thick flake exposed under laboratory conditions.

Unlike GaS, GaSe is less stable in air, and a peak associated with the α-Se phase appears after 2 h of exposure^56–59^. We investigated the stability of the GaS_0.5_Se_0.5_ alloy, which is a mixed crystal of GaS and GaSe^52,56–59,88,89^. The Raman spectrum shows three peaks, which correspond to the A_1g_(1), A_1g_(2), and E_g_(1) modes, positioned as reported by Gasanly et al.^88^. The thickness-dependent Raman spectra for GaS_0.5_Se_0.5_ and the oxidation studies are illustrated in Fig. 13.

A comparison of the stabilities of GaS and GaS_0.5_Se_0.5_ under ambient conditions indicates higher resistance to the degradation of the GaS material. This assumption is visible from the comparison of the decrease in the intensity of the Raman modes for both materials. Therefore, the GaS_0.5_Se_0.5_ alloy is less resistant; after three weeks of exposure to ambient air, almost all Raman modes are weaker and difficult to detect (Fig. 13b). The lower resistance of the GaS_0.5_Se_0.5_ compound is attributed to the addition of Se atoms. Results from the EDS analysis suggest a stronger oxidation of this material, as shown in Figs. S20–S21 in ESM.

## Discussion

The degradation of optical properties in the laboratory atmosphere is assumed to be related to the oxidation process for all studied crystals. The oxidation mechanism of LMs can be divided into two types. The first type relies on material oxidation from only the top. The second type involves oxidation from the top and from the sides, which is very important for small flakes. Another important factor that affects the timing and mechanism of oxidation is the thickness of the layered materials. Therefore, oxidation is more important for thin flakes because it accounts for a greater percentage of the total thickness of the flakes.

**Figure 14:**
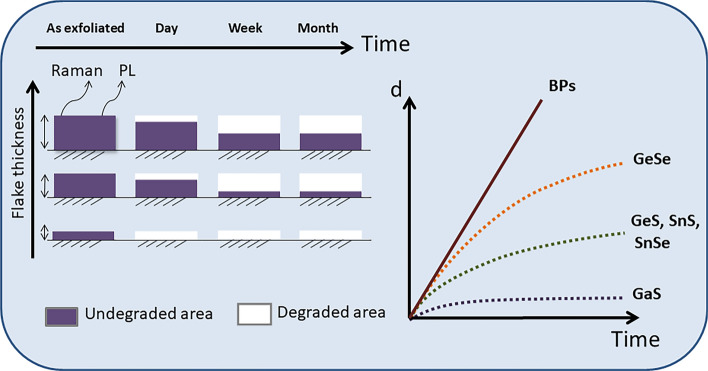
Thickness dependent oxidation mechanism of studied monochalcogenides.

Oxidation was visible for most of the studied layered materials, and it was observed with the help of selected markers. A summary of the Raman and PL studies on the degradation of LMs based on oxidation is shown in Fig. 14. An oxide layer emerged on thin exfoliated flakes after exposure to ambient air. For thicker flakes, the oxide layer works as the passivation layer and provides a protective coating against further degradation.

According to oxidation mechanism, the first stage includes the oxidation of LMs attributed to hybridisation and chemisorption with O_2_ molecules on the flake surface. The second stage relies on the formation of the passivation layer on the surface during exposure to ambient conditions. This passivation layer can protect the material from further degradation; however, this depends on the material. Black phosphorus (BPs) is an example of a material where the oxidized layer does not protect the crystal from further oxidation.

Our results indicate that higher stability (up to three weeks) was observed for the GaS material while monochalcogenides with the Se content were the most reactive. Furthermore, the SEM results indicated surface protrusions after GeSe was exposed to ambient (Table 2).

**Table 2:** Level of ambient degradation for the layered LMs four weeks after exfoliation.

Material	Low degradation	Medium degradation	Strong degradation
GeS		X	
GeSe			X
SnS		X	
SnSe		X	
GaS	X		
GaS_0.5_Se_0.5_		X	

## Conclusions

We analyzed the stability of group III and group IV LMs. All materials used in this study were obtained by mechanical exfoliation from bulk crystals. The degradation of the exfoliated flakes of LMs in air was investigated using Raman spectroscopy, PL, electron microscopy, and EDS. For all studied materials, the oxygen on the surface was confirmed by EDS. For thicker flakes, the oxide layer acts as the passivation and protective coating. However, for atomically thin flakes, the oxidation process is very important and cannot be neglected in optical characterization and device fabrication. We observed that LMs are more challenging to exfoliate than transition metal dichalcogenides. Further, their stability with time is worse than that of transition metal dichalcogenides such as MoS_2_, MoSe_2_, WS_2_, or WSe_2_. Therefore, the production of devices with LMs requires controlling the environmental conditions (e.g., argon or nitrogen atmosphere) and a layer protecting the material against the oxidation process.

### Supplementary Information

Supplementary Video 1.

Supplementary Video 2.

Supplementary Video 3.

Supplementary Information 1.

Supplementary Figure 1.

Supplementary Figure 2.

Supplementary Figure 3.

Supplementary Figure 4.

Supplementary Figure 5.

Supplementary Figure 6.

Supplementary Figure 7.

Supplementary Figure 8.

Supplementary Figure 9.

Supplementary Figure 10.

Supplementary Figure 11.

Supplementary Figure 12.

Supplementary Figure 13.

Supplementary Figure 14.

Supplementary Figure 15.

Supplementary Figure 16.

Supplementary Figure 17.

Supplementary Figure 18.

Supplementary Figure 19.

Supplementary Figure 20.

Supplementary Figure 21.

## Data Availability

The data presented in this study are available upon reasonable request from the corresponding author.
