# Supplementary material for: Stability of mechanically exfoliated layered monochalcogenides under ambient conditions
Source: Sci Rep. 2023 Nov 4;13:19114. doi: 10.1038/s41598-023-46092-1 (PMC10625602; doi:10.1038/s41598-023-46092-1)
Supplement: Supplementary file 4 — Supplementary Information 1. [file 41598_2023_46092_MOESM4_ESM.docx]

**Electronic Supplementary Material**

| **Stability of mechanically exfoliated layered monochalcogenides under ambient conditions** |
| --- |
| Daria Hlushchenko^1,2,^*, Anna Siudzinska^1^, Joanna Cybinska^1,3^, Malgorzata Guzik^1,3^, Alicja Bachmatiuk^1^, Robert Kudrawiec^1,2^  *^1^Lukasiewicz Research Network, PORT Polish Center for Technology Development, Stablowicka 147, 54-066, Wroclaw, Poland;*  *^2^Wroclaw University of Science and Technology, Faculty of Fundamental Problems of Science and Technology, ul. Wybrzeze Wyspianskiego 27, 50-370 Wroclaw, Poland*  *^3^University of Wroclaw, Faculty of Chemistry, ul. F. Joliot-Curie 14, 50-383, Wroclaw, Poland*  **Correspondence should be addressed to D.H. and R.K. (email:* [*daria.hlushchenko@pwr.edu.pl*](mailto:daria.hlushchenko@pwr.edu.pl)*, robert.kudrawiec@pwr.edu.pl)* |


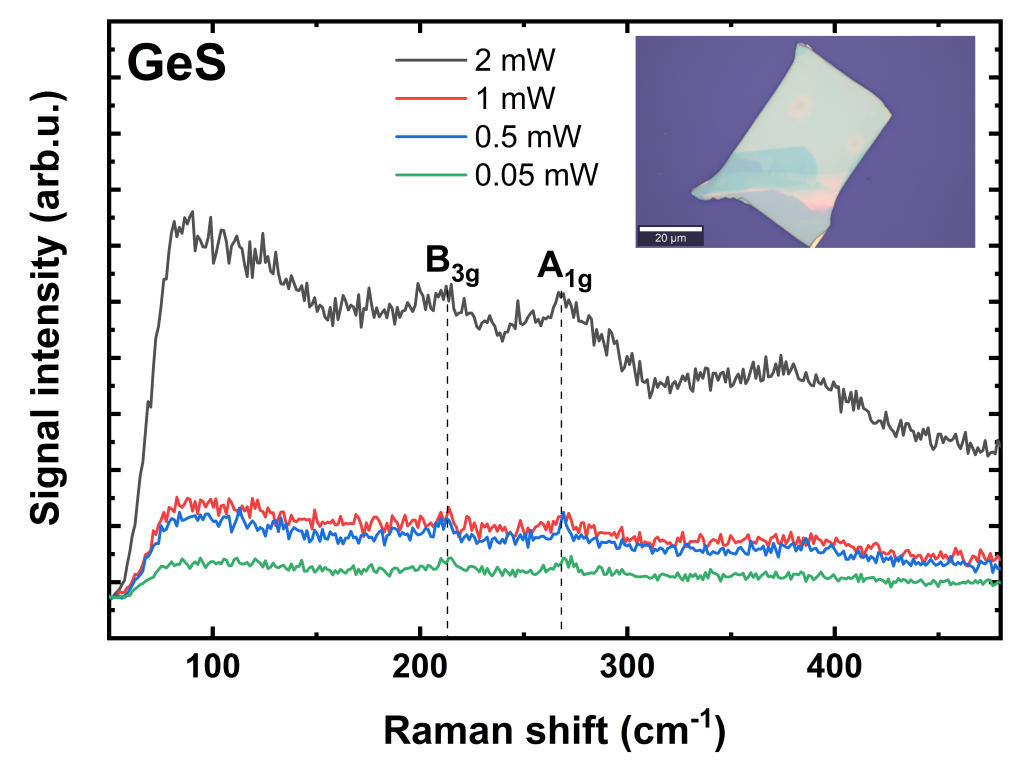


**Figure S1** Raman spectra for freshly exfoliated GeS flake examining the selection of laser power


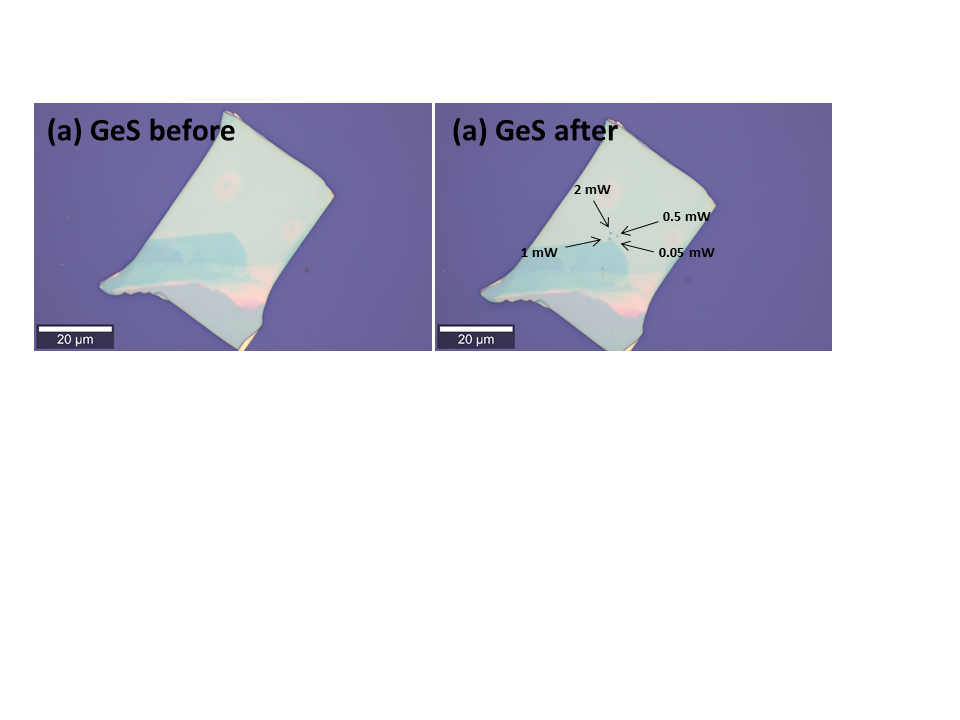


**Figure S2** Images for GeS flake: (a) before Raman measuring; (b) after Raman measuring. On flake the four laser power was tested: 0.05 mW (after the measurement, no visible changes were observed on the flake surface); for another powers 0.5-2 mW the marks form laser were remained after the measurement.


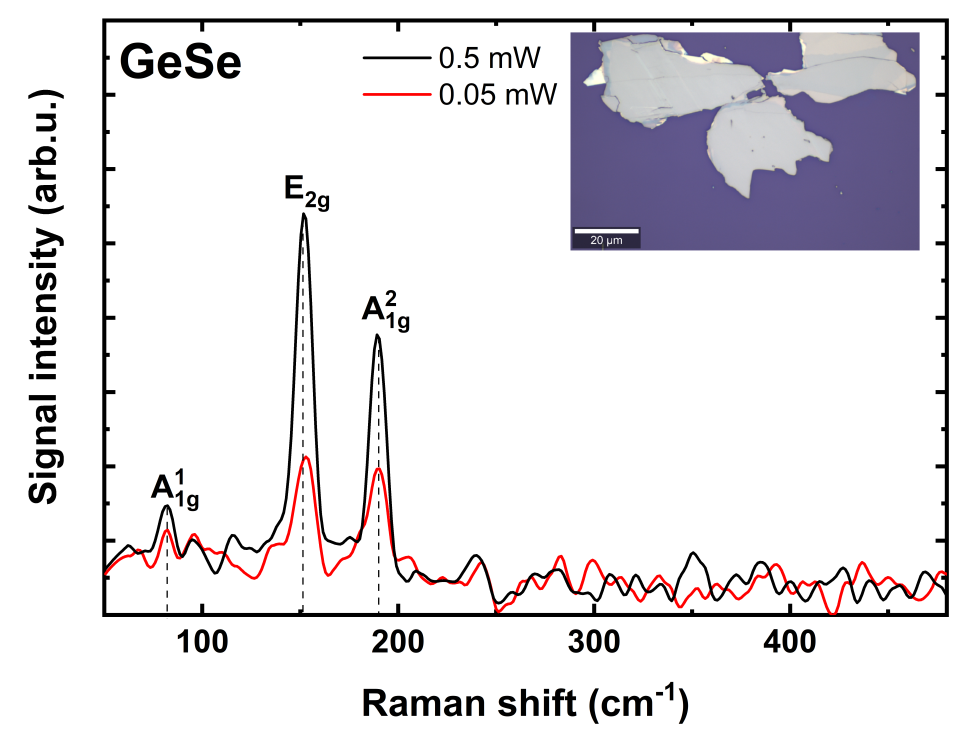


**Figure S3** Raman spectra for freshly exfoliated GeSe flake examining the selection of laser power


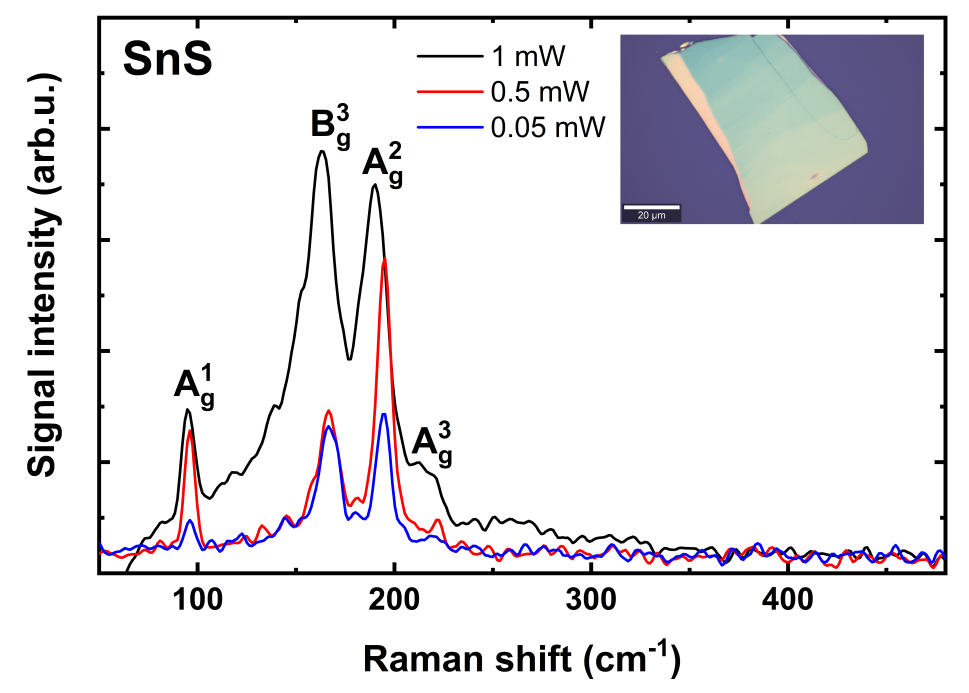


**Figure S4** Raman spectra for freshly exfoliated SnS flake examining the selection of laser power


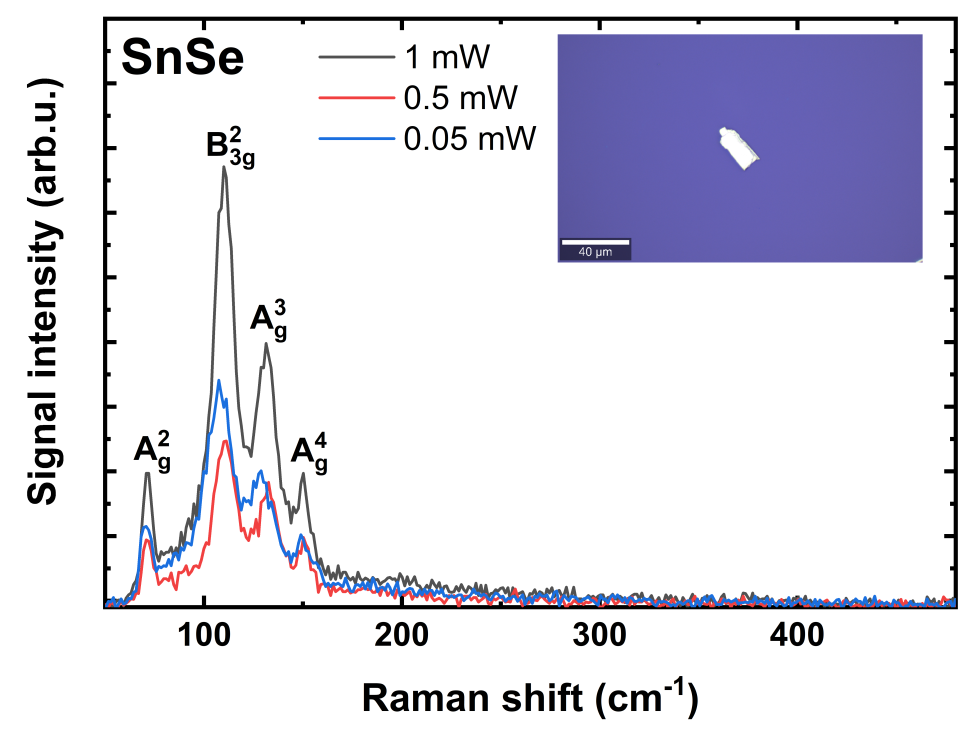


**Figure S5** Raman spectra for freshly exfoliated SnSe flake examining the selection of laser power


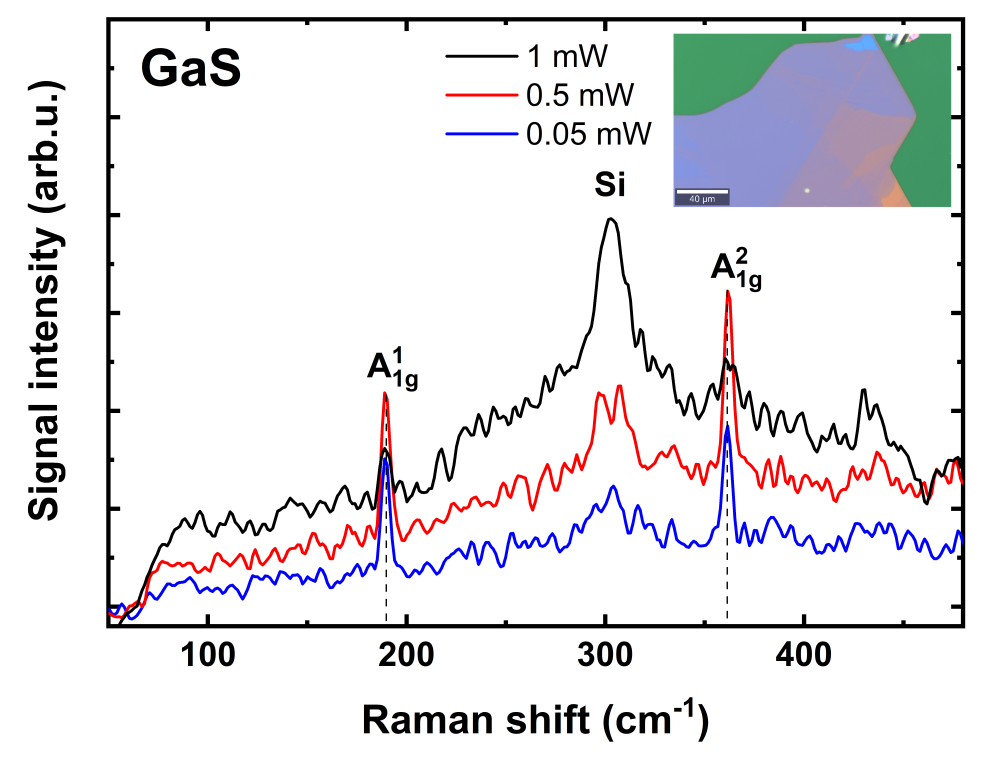


**Figure S6** Raman spectra for freshly exfoliated GaS flake examining the selection of laser power


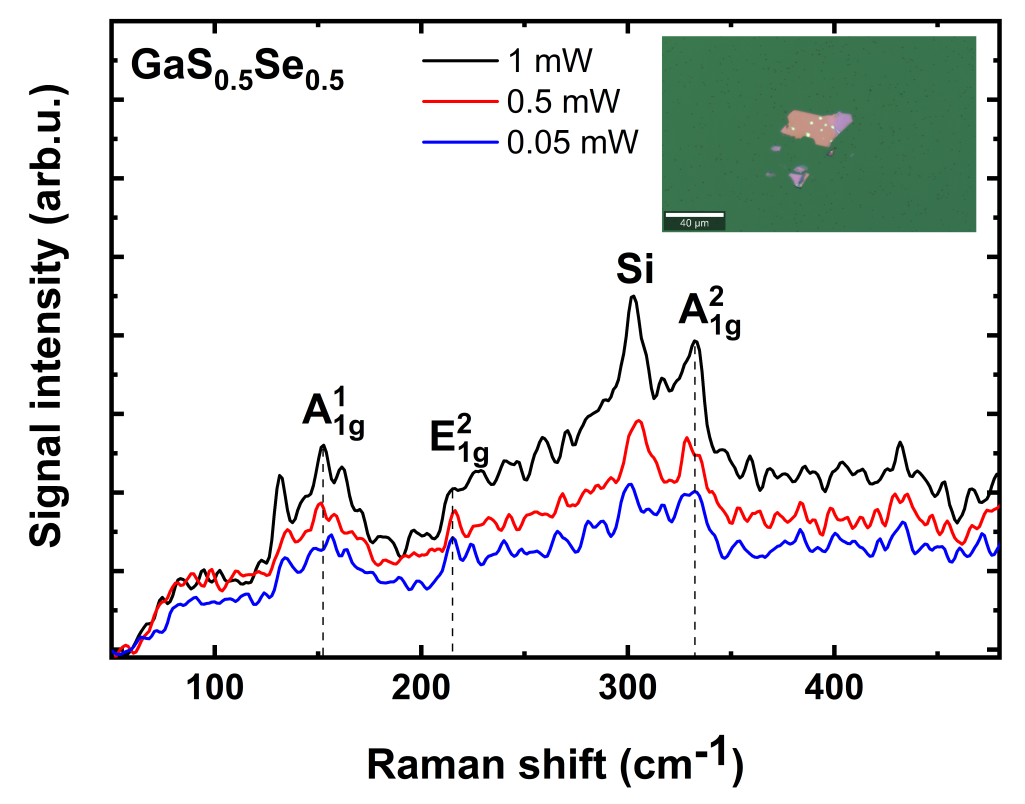


**Figure S7** Raman spectra for freshly exfoliated GaS_0.5_Se_0.5_ flake examining the selection of laser power

Images from optical microscope of freshly exfoliated monolayers (GaS (a), GaS_0.5_Se_0.5_ (b), GeS (c), SnSe (d)).


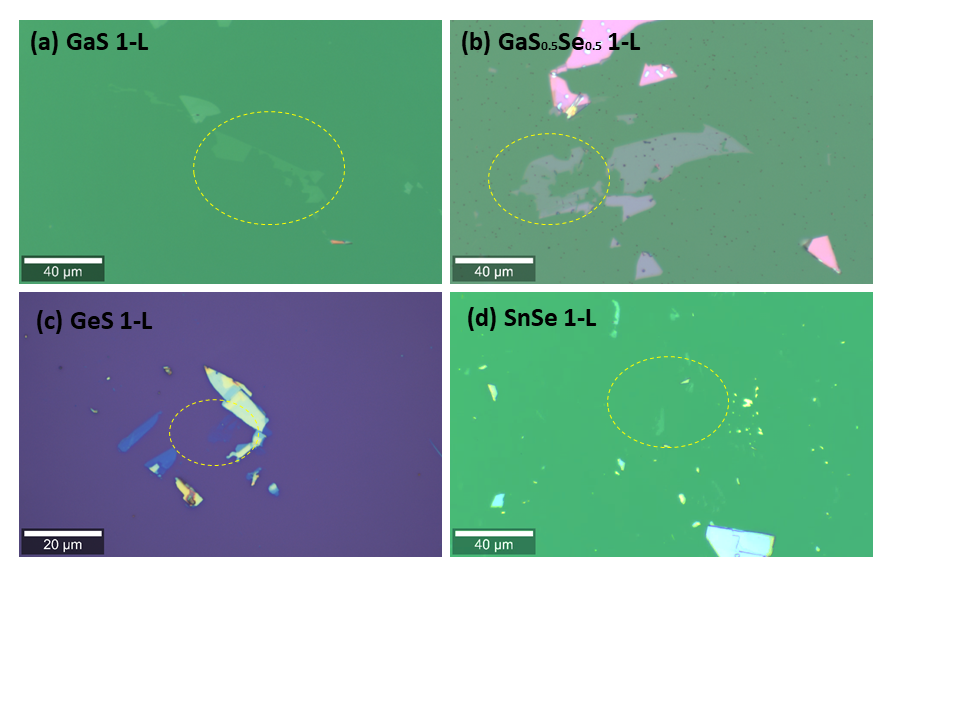


**Figure S8** Images of freshly exfoliated monochalcogenides 1-Layers: (a) GaS 1-L on Si_3_N_4_/Si; (b) GaS_0.5_Se_0.5_ 1-L on Si_3_N_4_/Si; (c) GeS 1-L on SiO_2_/Si; (d) SnSe 1-L on Si_3_N_4_/Si.

**
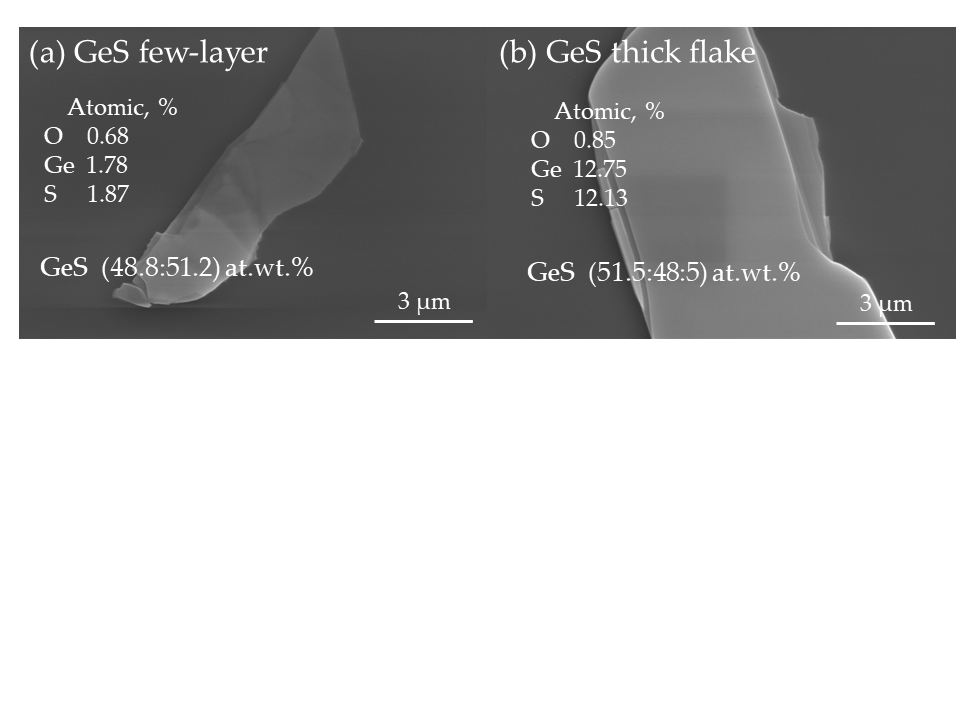
**

**Figure S9** SEM images of oxidized GeS flakes on Si_3_N_4_/Si after three weeks at ambient conditions: (a) Few-layer GeS; (b) Thick GeS flake. Results from the EDS flake analysis are presented on each image. Substrate Si_3_N_4_/Si

**
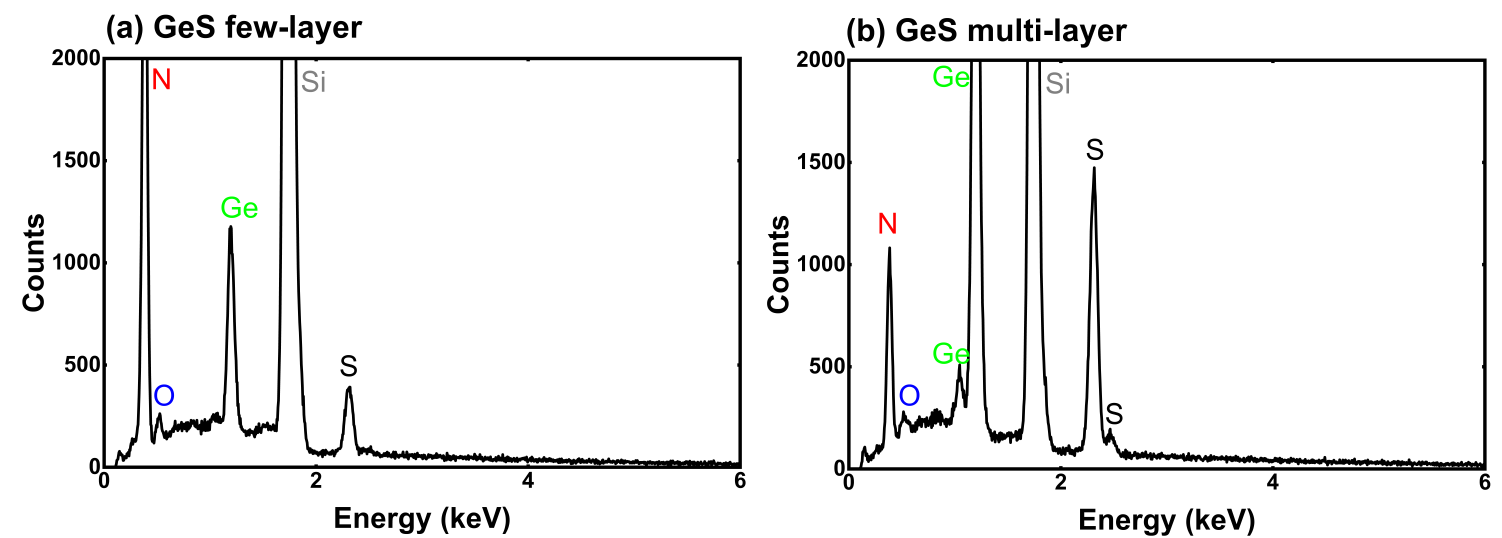
**

**Figure S10** EDS spectra for oxidized GeS flakes on Si_3_N_4_/Si after three weeks at ambient conditions: (a) Few-layer GeS; (b) Thick GeS flake.

**
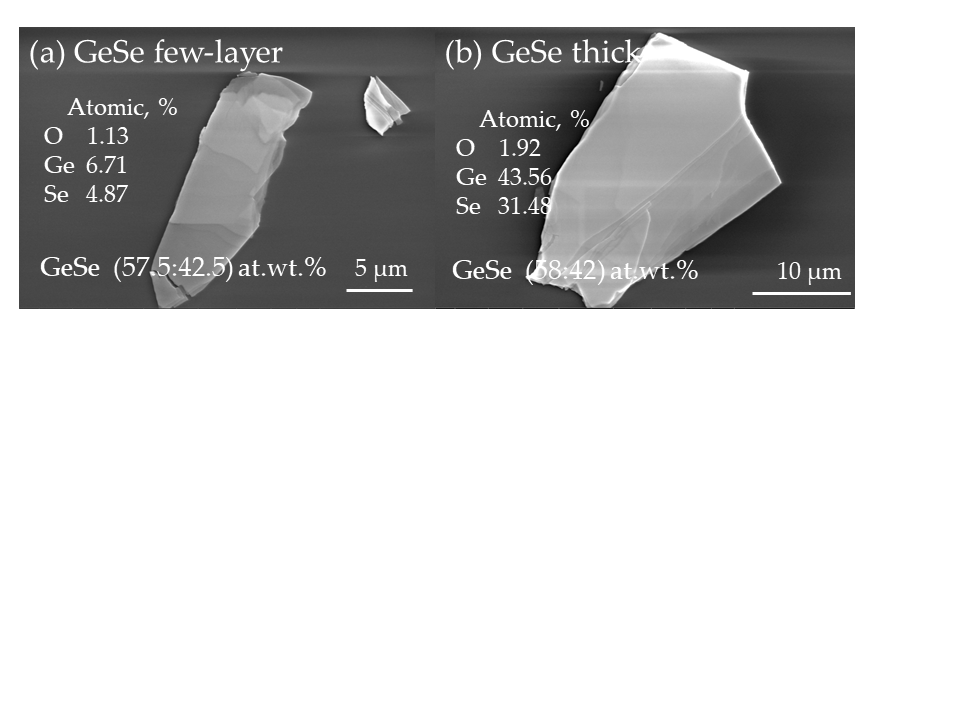
**

**Figure S11** SEM images of oxidized GeSe flakes on Si_3_N_4_/Si after three weeks at ambient conditions: (a) Few-layer GeSe; (b) Thick GeSe flake. Results from the EDS flake analysis are presented on each image. Substrate Si_3_N_4_/Si


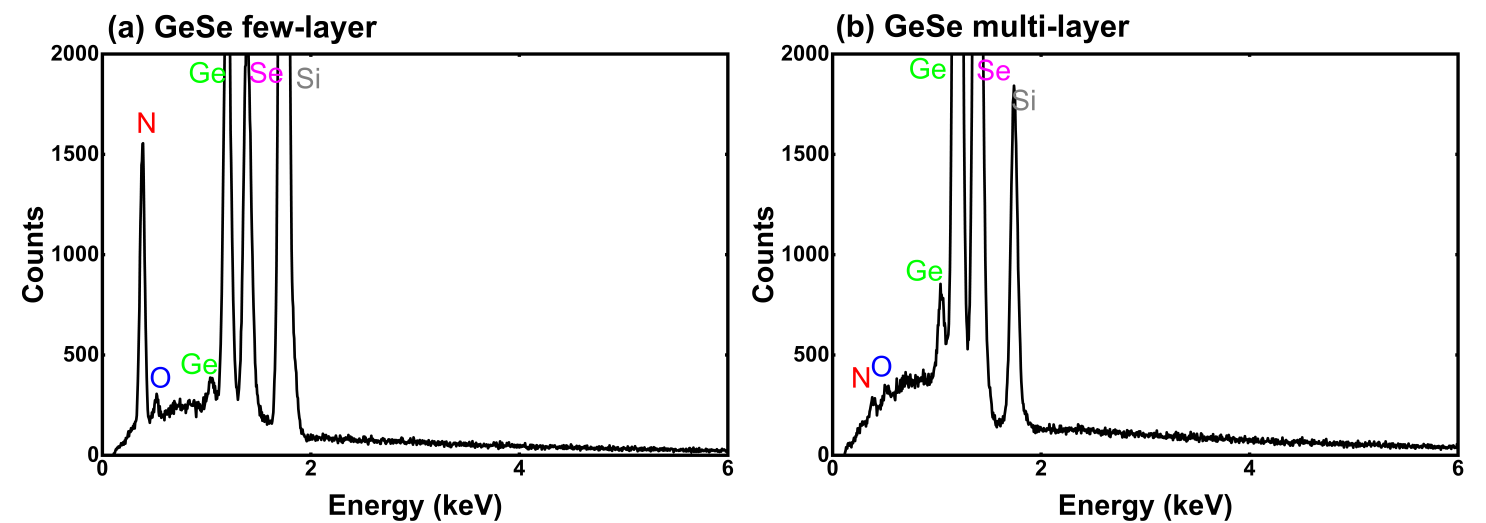


**Figure S12** EDS spectra for oxidized GeSe flakes on Si_3_N_4_/Si after three weeks at ambient conditions: (a) Few-layer GeSe; (b) Thick GeSe flake.

**
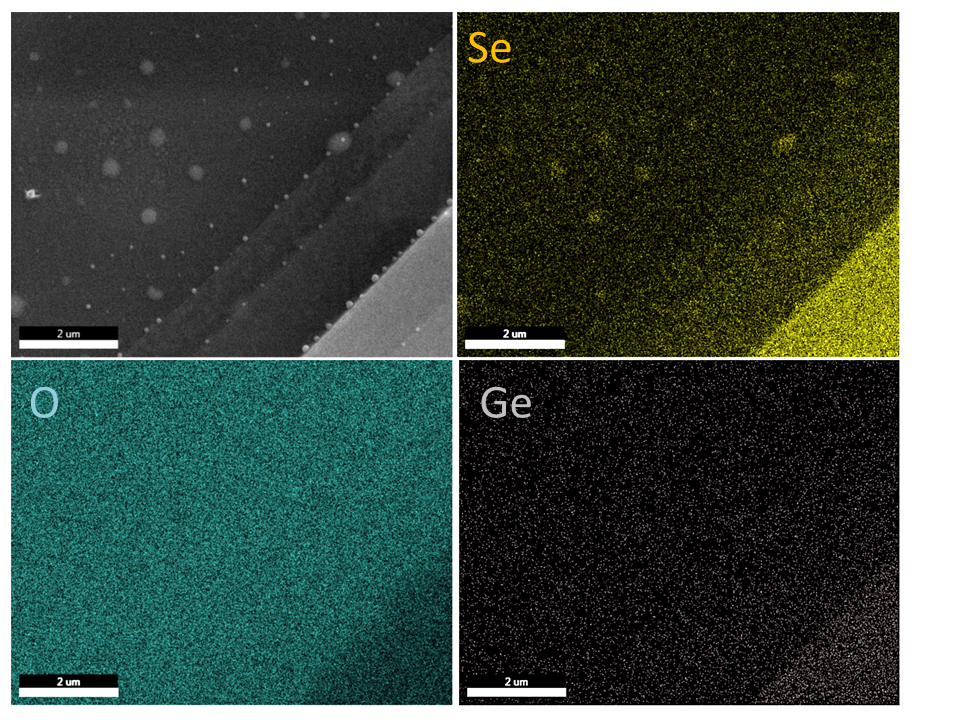
**

**Figure S13** EDS map for protrusions on oxidised GeSe flake. The EDX map confirms the Selenium (Se) content on the observed protrusions.

**
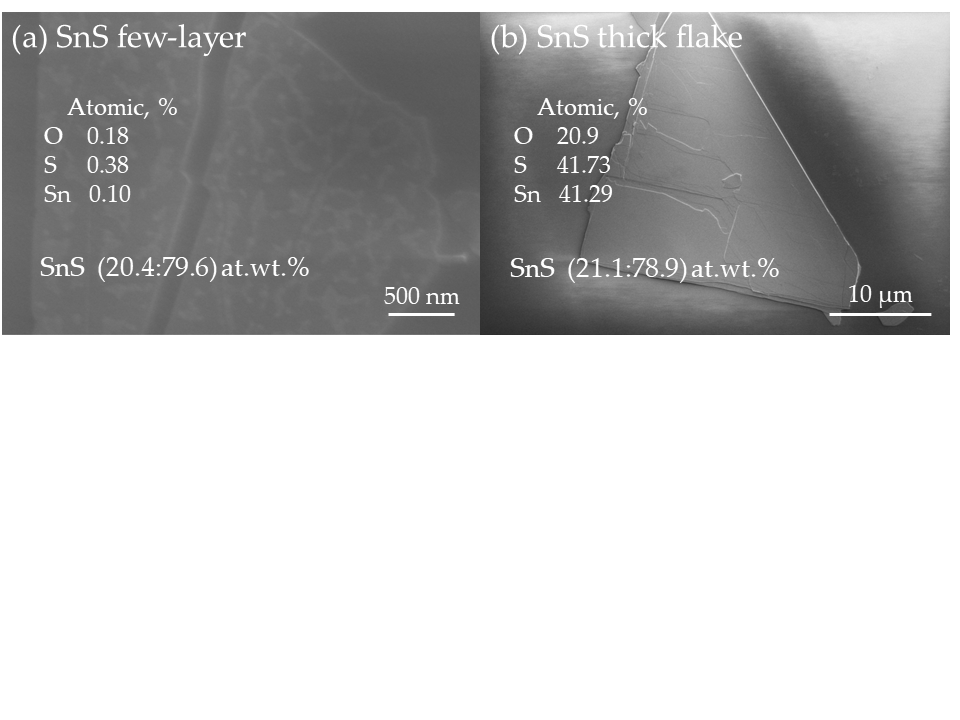
**

**Figure S14** SEM images of oxidized SnS flakes on Si_3_N_4_/Si after three weeks at ambient conditions: (a) Few-layer SnS; (b) Thick SnS flake. Results from the EDS flake analysis are presented on each image.


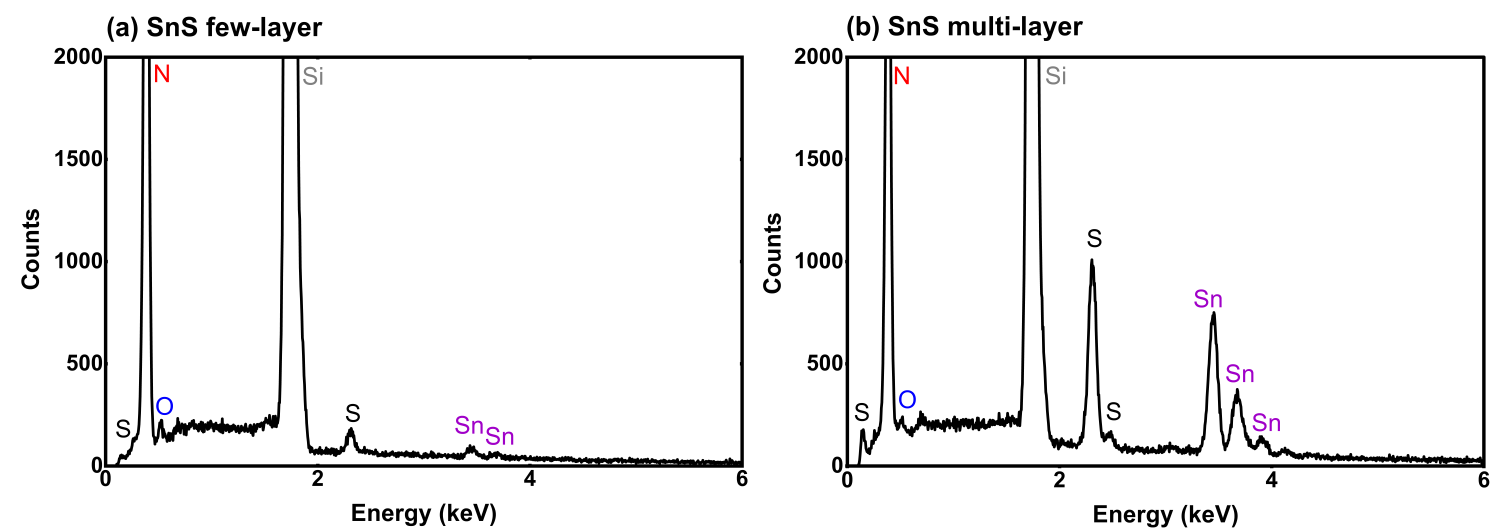


**Figure S15** EDS spectra for oxidized SnS flakes on Si_3_N_4_/Si after three weeks at ambient conditions: (a) Few-layer SnS; (b) Thick SnS flake.

**
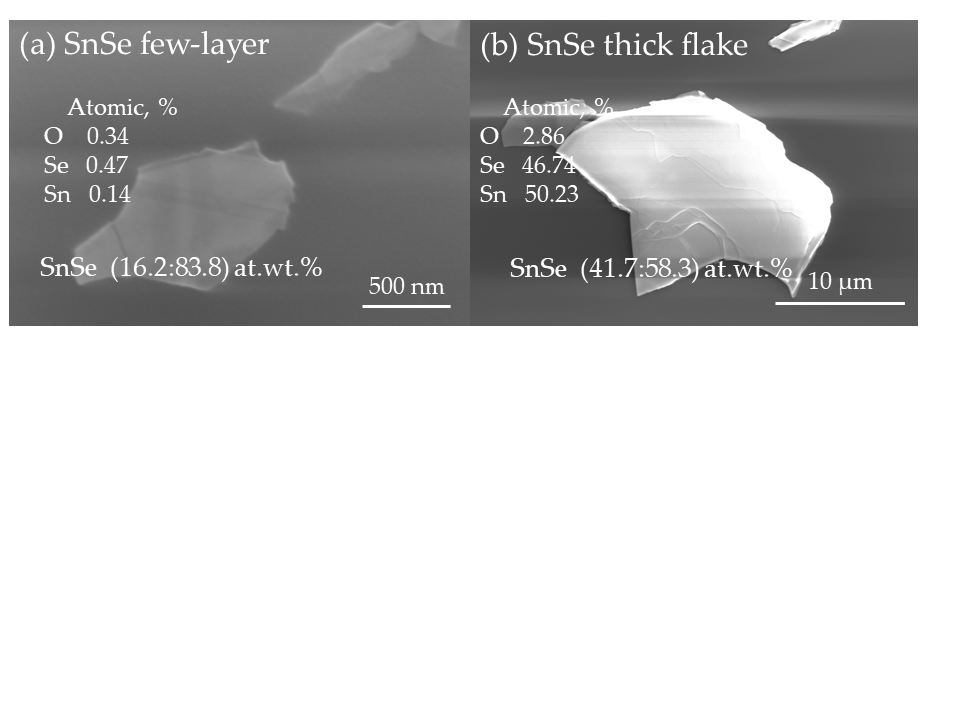
**

**Figure S16** SEM images of oxidized SnSe flakes on Si_3_N_4_/Si after three weeks at ambient conditions: (a) Few-layer SnSe; (b) Thick SnSe flake. Results from the EDS flake analysis are presented on each image.


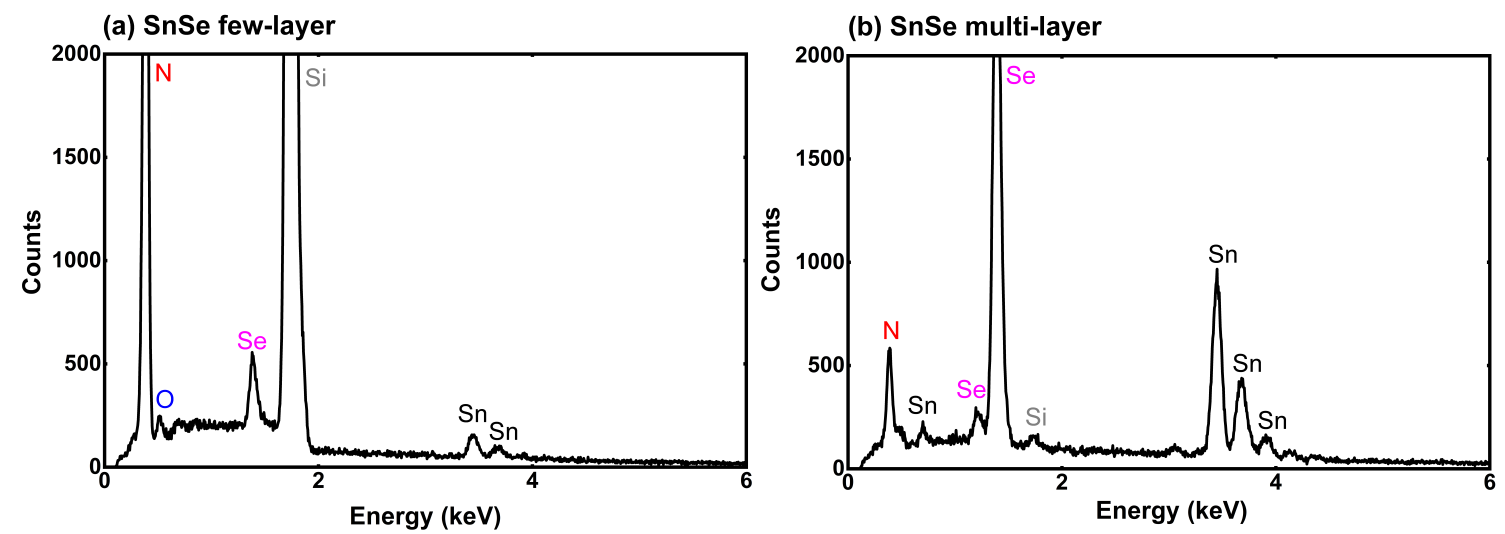


**Figure S17** EDS spectra for oxidized SnSe flakes on Si_3_N_4_/Si after three weeks at ambient conditions for: (a) Few-layer SnSe; (B) Multi-layer SnSe.

**
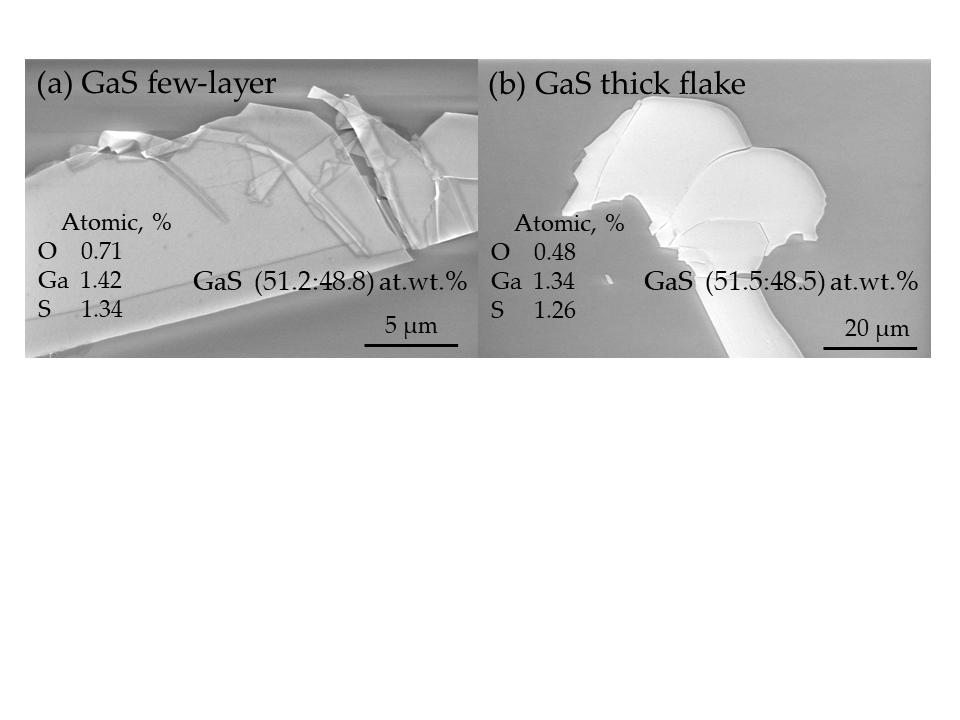
**

**Figure S18** SEM images of oxidized GaS flakes on Si_3_N_4_/Si after three weeks at ambient conditions: (a) Few-layer GaS layer; (b) Thick GaS flake. Results from the EDS flake analysis are presented on each image.


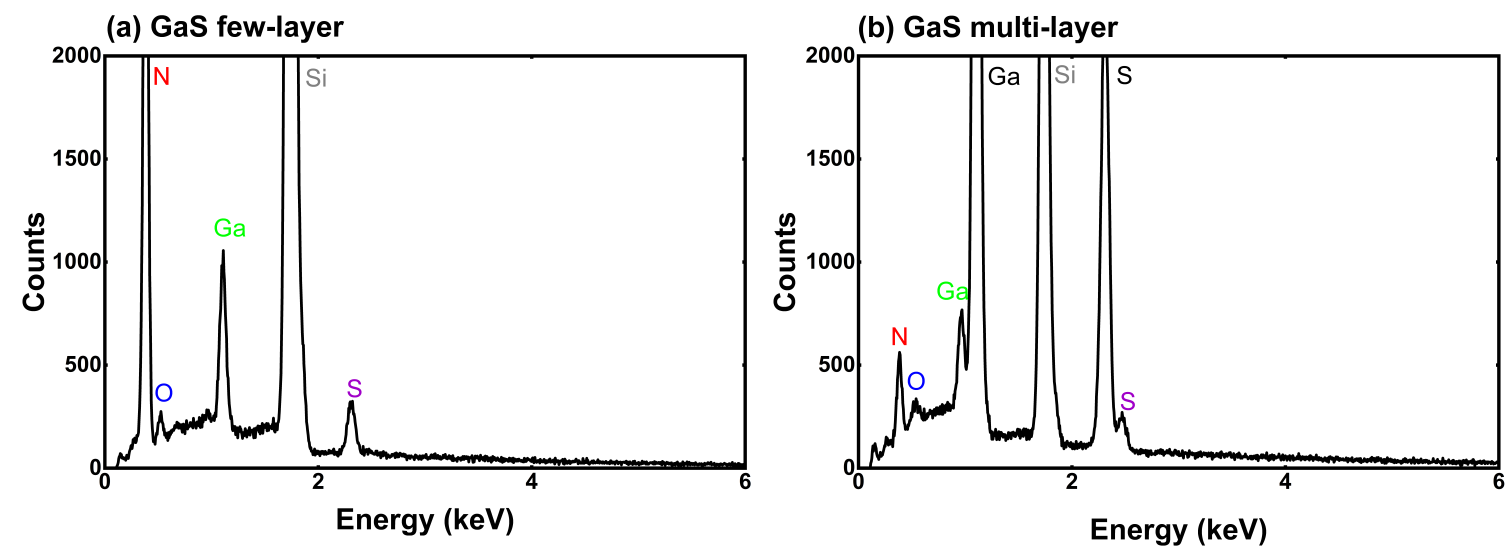


**Figure S19** EDS spectra for oxidized GaS flakes on Si_3_N_4_/Si after three weeks at ambient conditions for: (a) Few-layer GaS; (b) Multi-layer GaS.

**
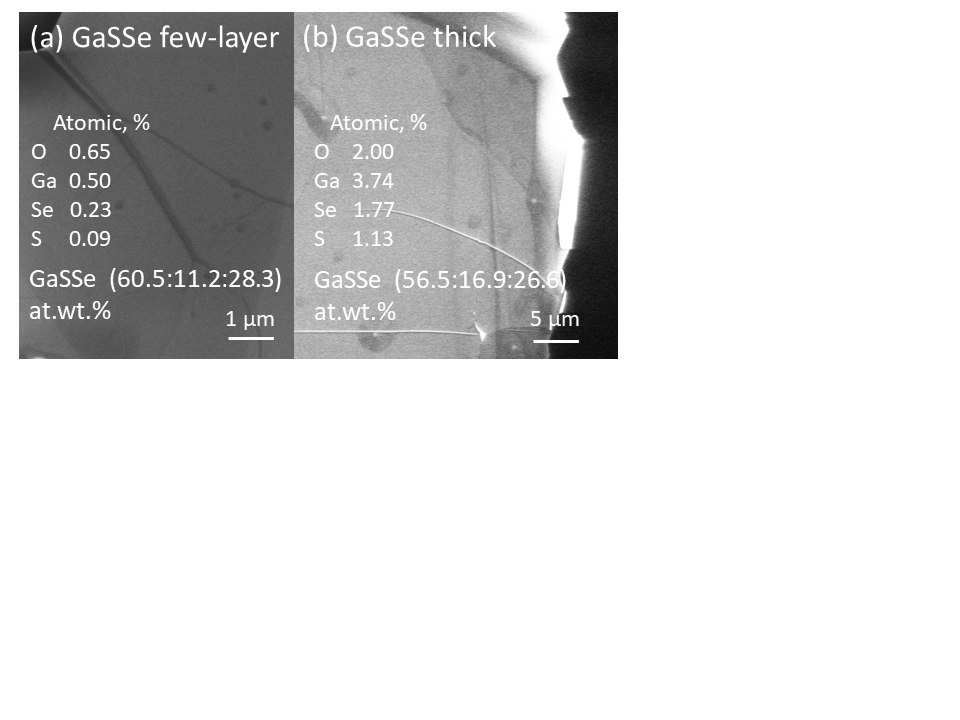
**

**Figure S20** SEM images of oxidized GaS_0.5_Se_0.5_ flakes on Si_3_N_4_/Si after three weeks at ambient conditions: (a) Few-layer; (b) Thick flake. Results from the EDS flake analysis are presented on each image.

**
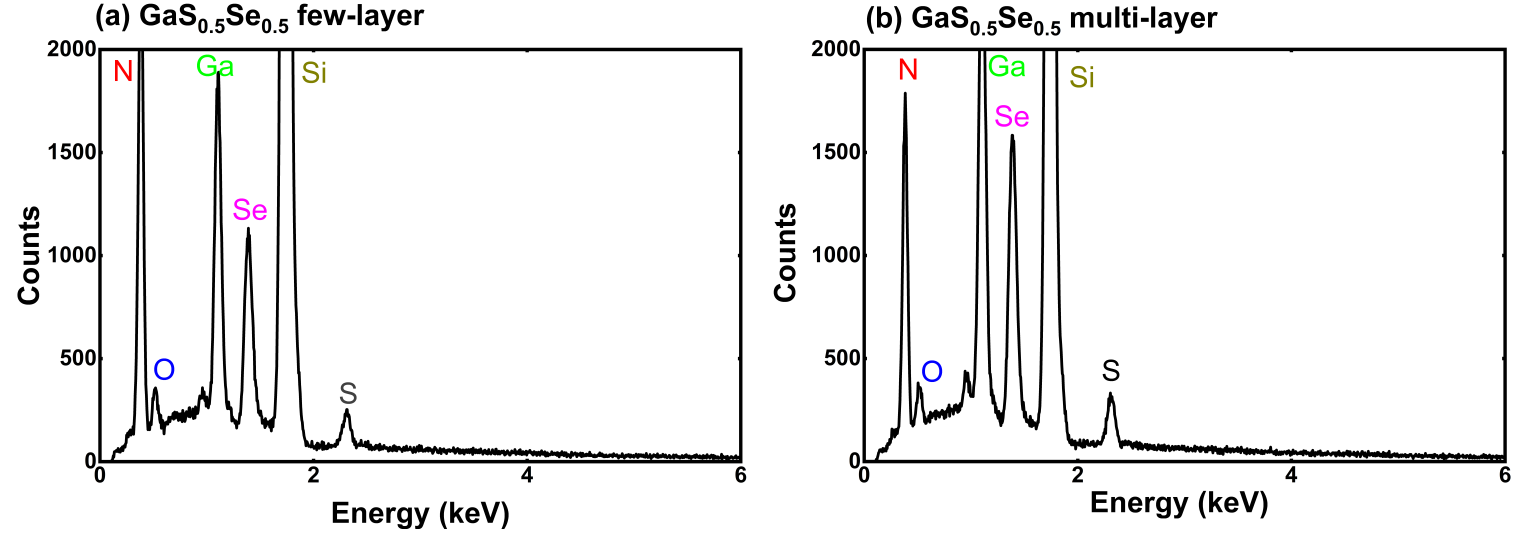
**

**Figure S21** EDS spectra for oxidized GaS_0.5_Se_0.5_ flakes on Si_3_N_4_/Si after three weeks at ambient conditions for: (a) Few-layer GaS_0.5_Se_0.5_; (b) Multi-layer GaS_0.5_Se_0.5._

**Animation 1** Oxidation of GeSe at the edge.

**Animation 2** Oxidation of GeS at the edge.
