# Supplementary figures and images for: Stability of mechanically exfoliated layered monochalcogenides under ambient conditions
Source: Sci Rep. 2023 Nov 4;13:19114. doi: 10.1038/s41598-023-46092-1 (PMC10625602; doi:10.1038/s41598-023-46092-1)

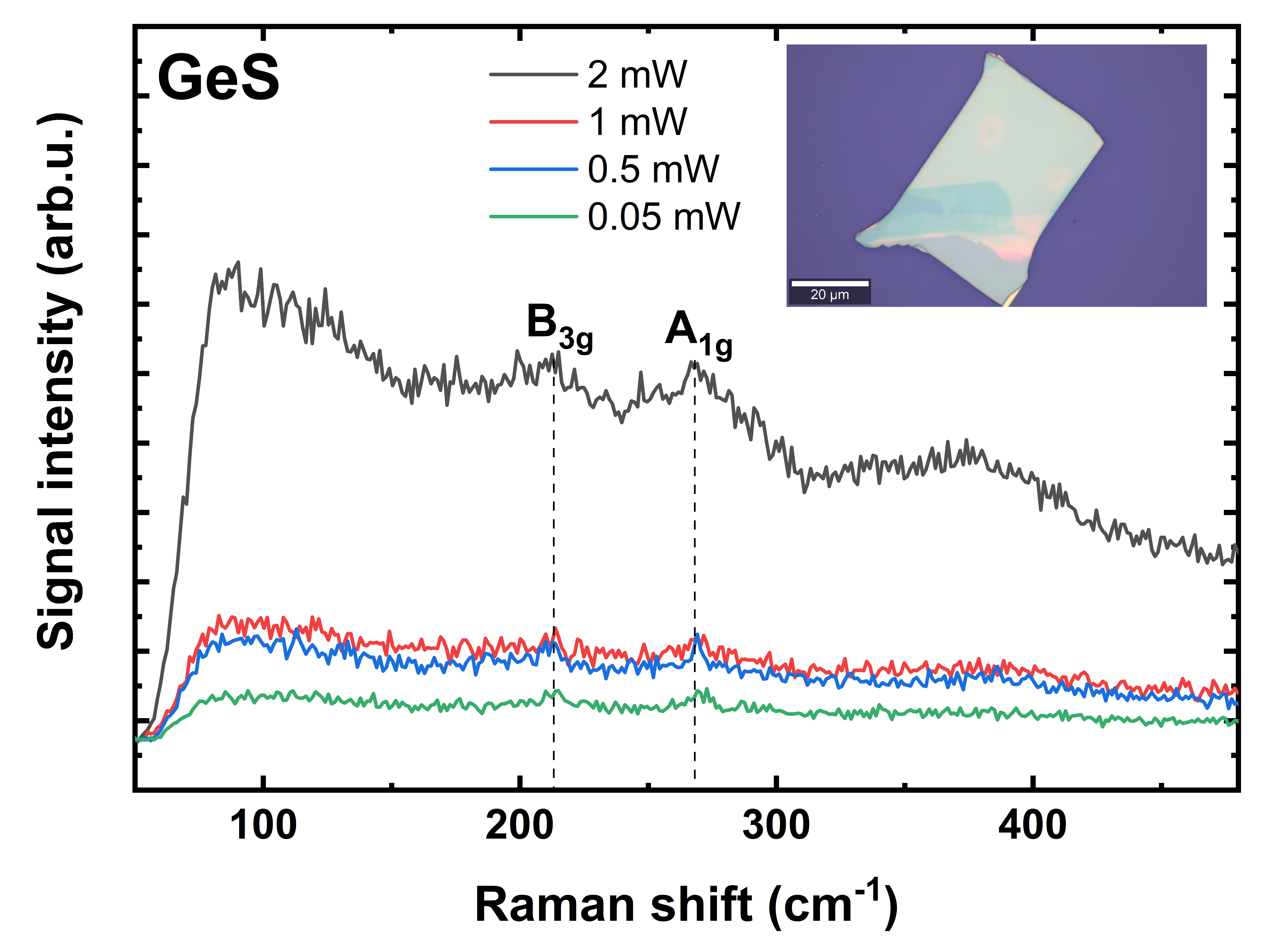

Supplement: Supplementary file 5 — Supplementary Figure 1. [file 41598_2023_46092_MOESM5_ESM.tif]

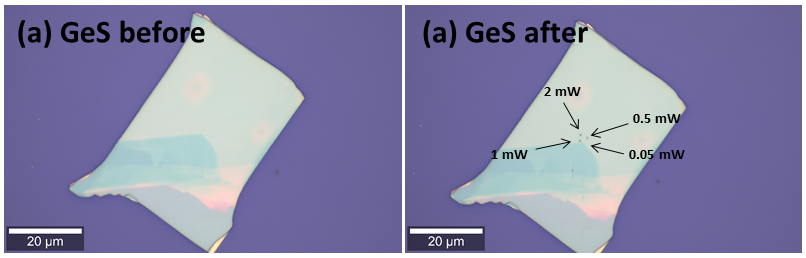

Supplement: Supplementary file 6 — Supplementary Figure 2. [file 41598_2023_46092_MOESM6_ESM.tif]

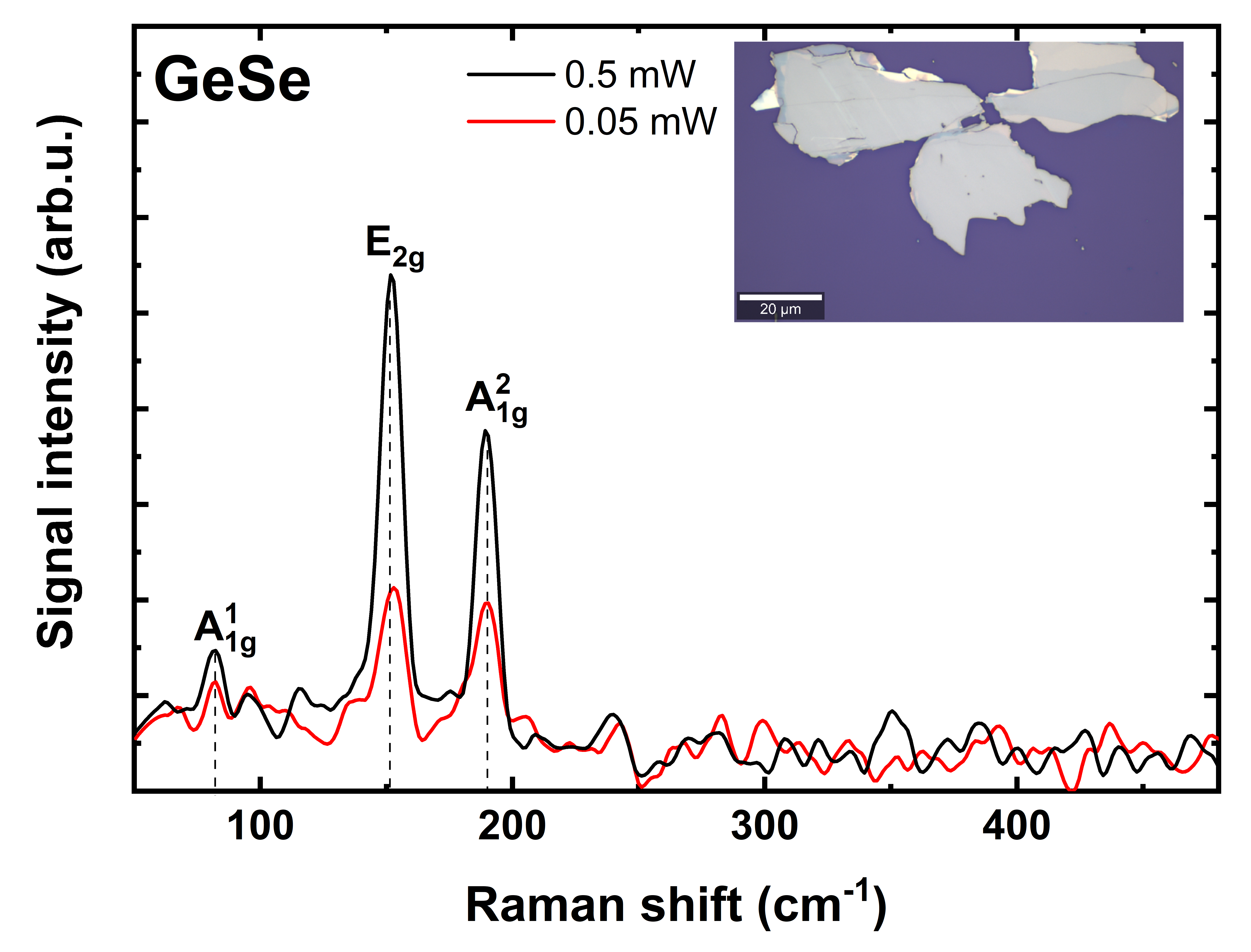

Supplement: Supplementary file 7 — Supplementary Figure 3. [file 41598_2023_46092_MOESM7_ESM.tif]

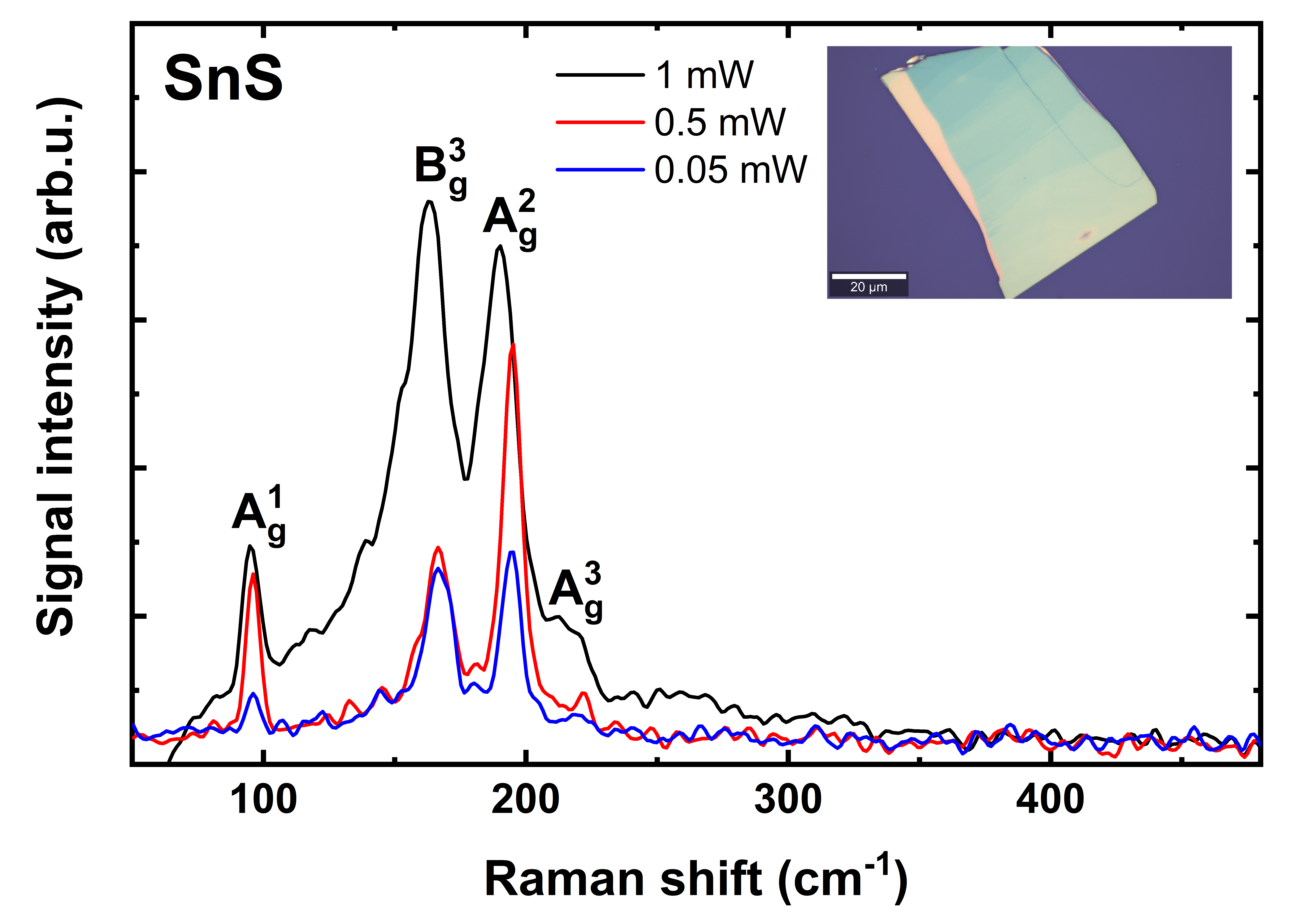

Supplement: Supplementary file 8 — Supplementary Figure 4. [file 41598_2023_46092_MOESM8_ESM.tif]

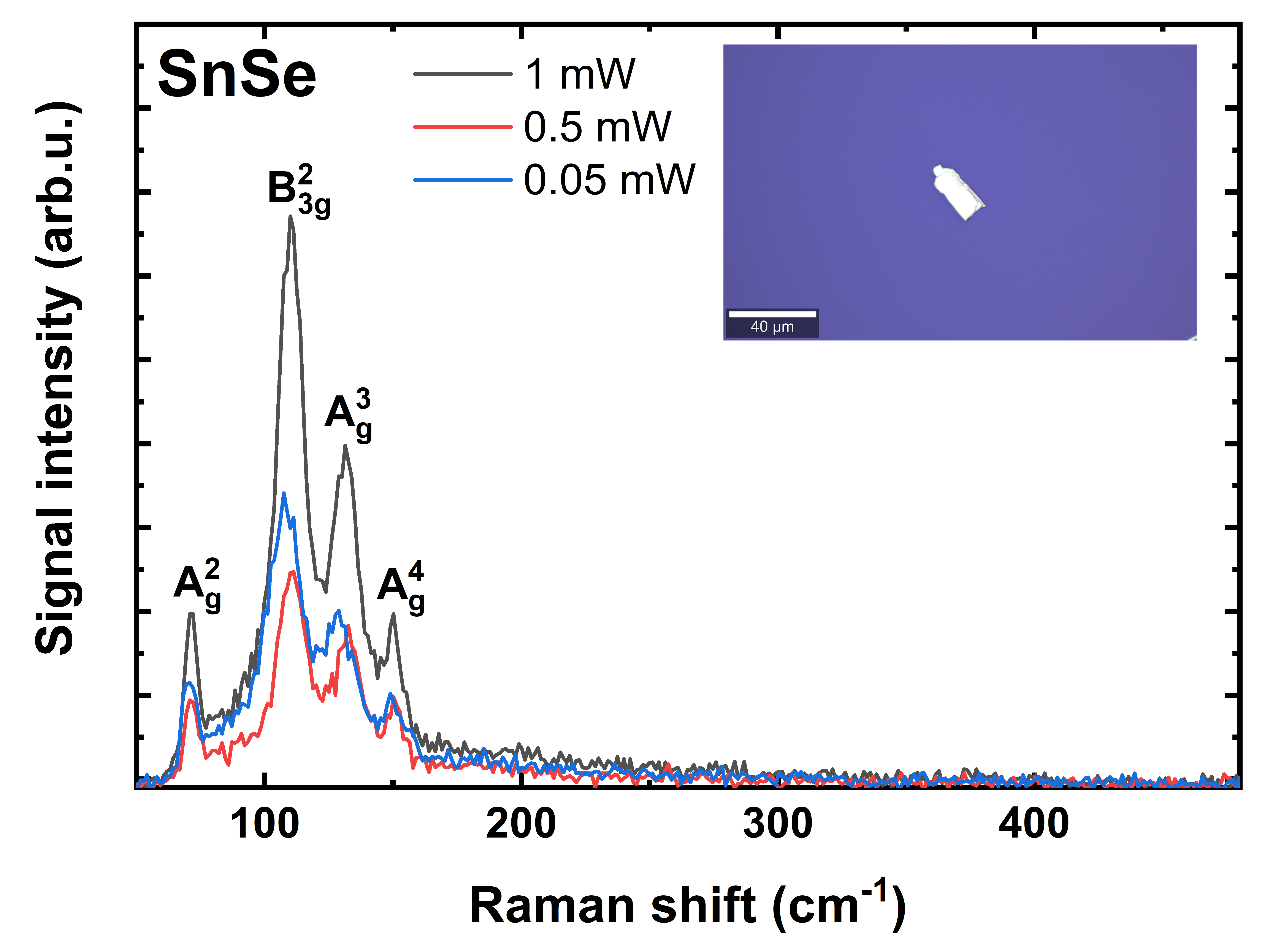

Supplement: Supplementary file 9 — Supplementary Figure 5. [file 41598_2023_46092_MOESM9_ESM.tif]

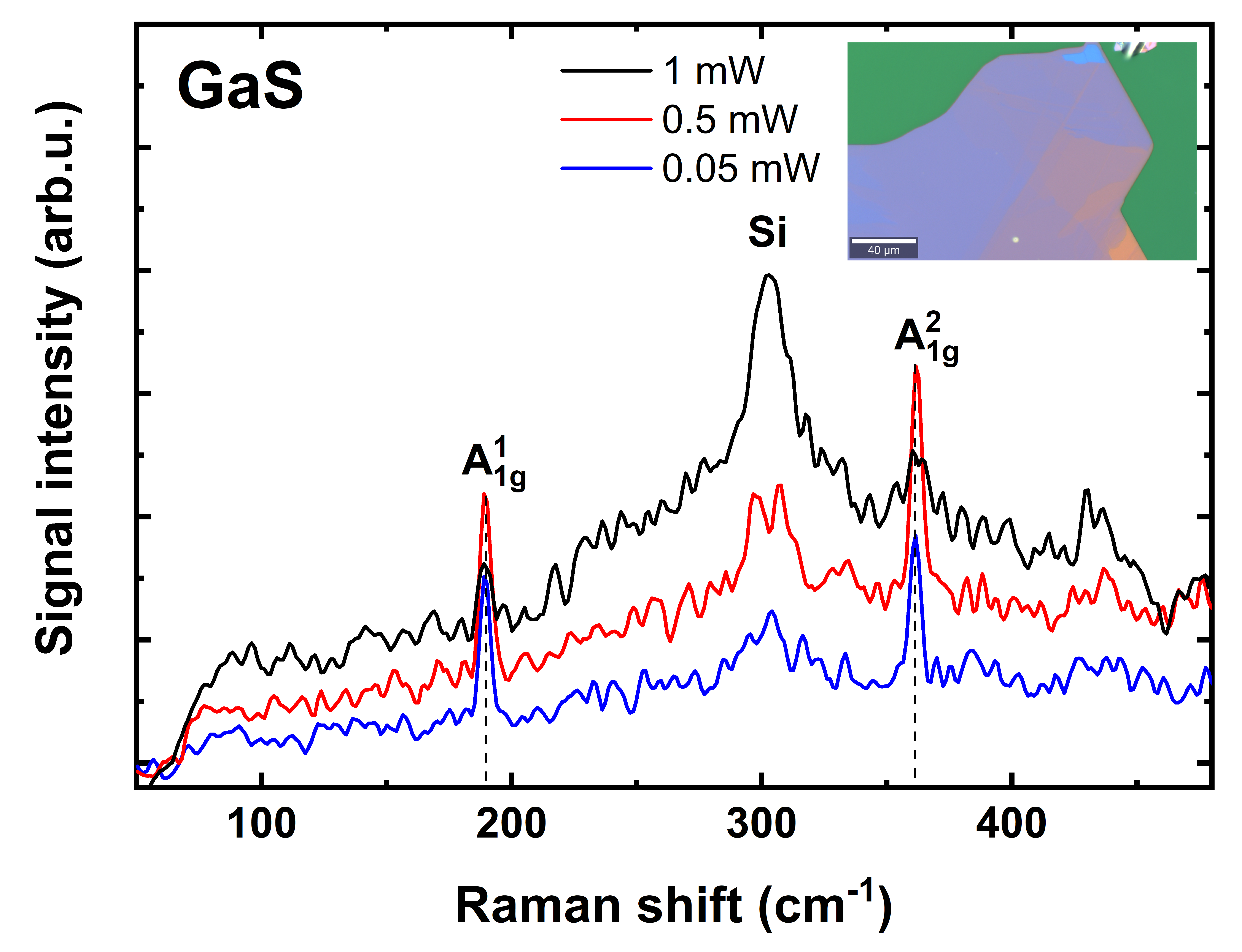

Supplement: Supplementary file 10 — Supplementary Figure 6. [file 41598_2023_46092_MOESM10_ESM.tif]

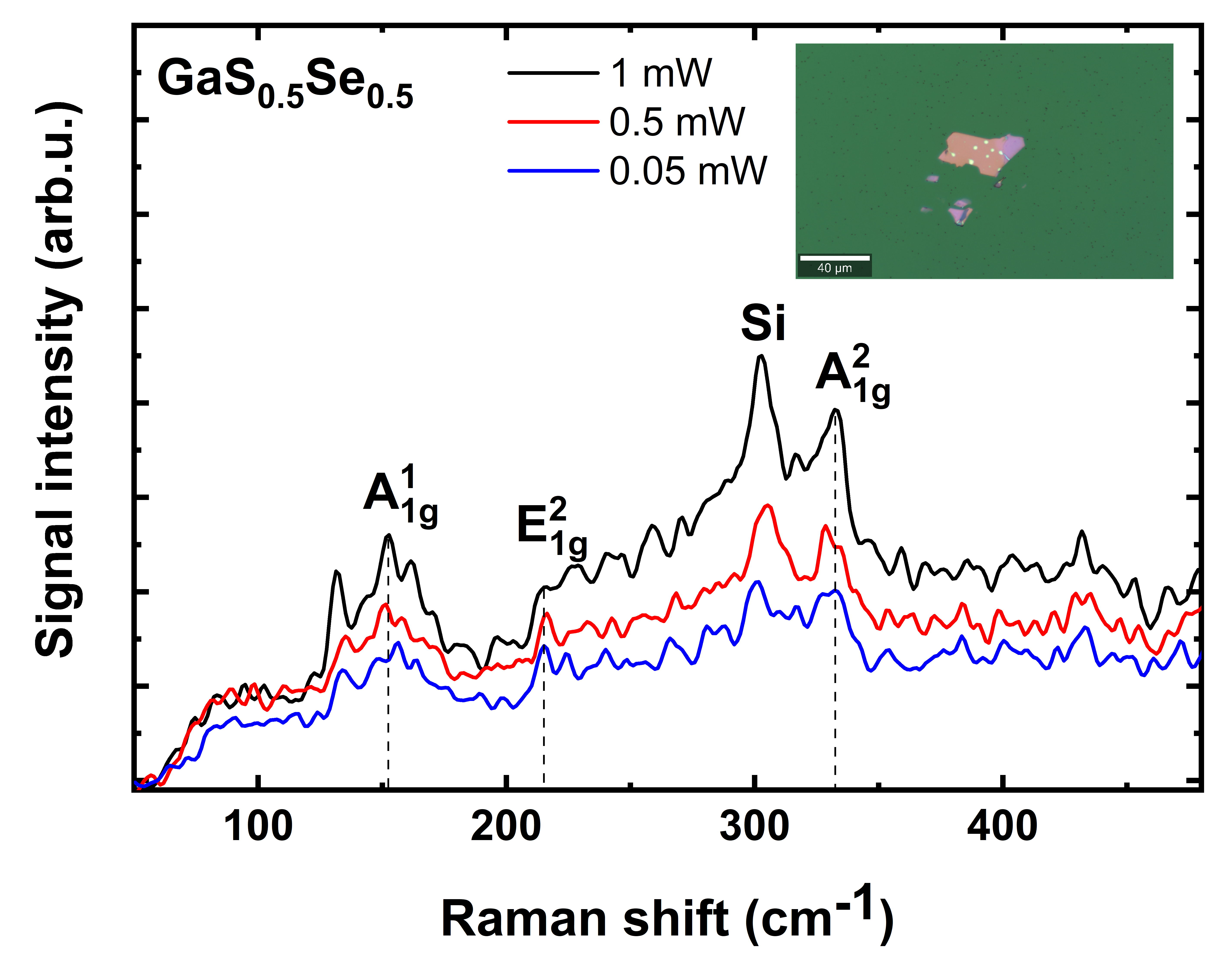

Supplement: Supplementary file 11 — Supplementary Figure 7. [file 41598_2023_46092_MOESM11_ESM.tif]

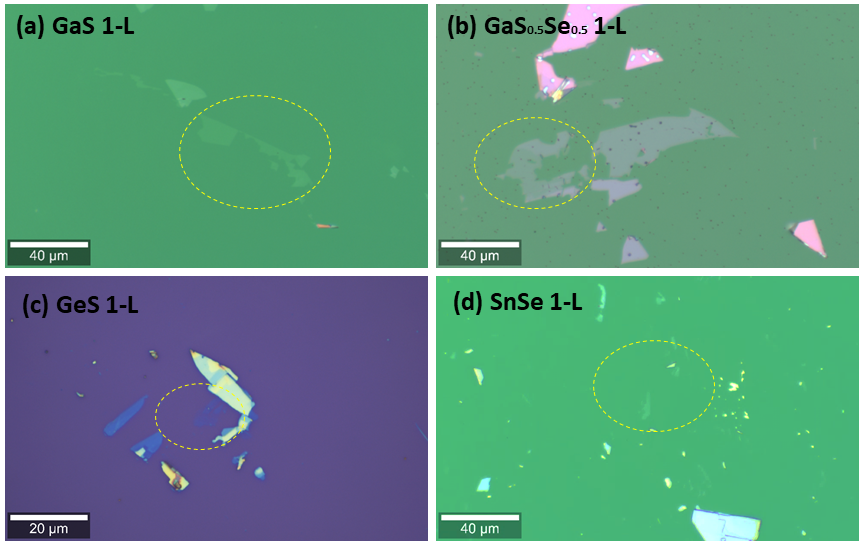

Supplement: Supplementary file 12 — Supplementary Figure 8. [file 41598_2023_46092_MOESM12_ESM.tif]

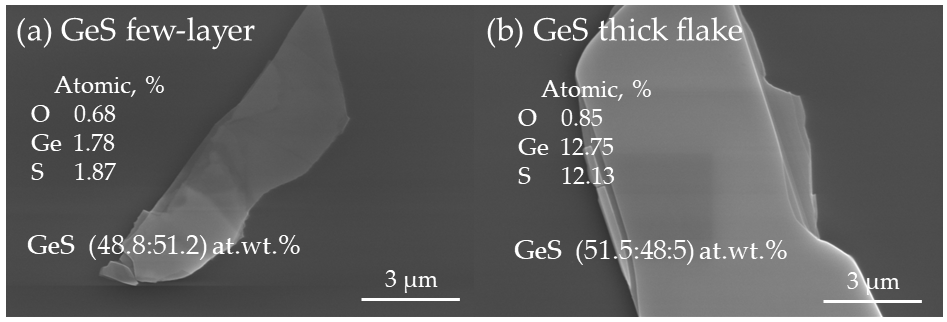

Supplement: Supplementary file 13 — Supplementary Figure 9. [file 41598_2023_46092_MOESM13_ESM.tif]

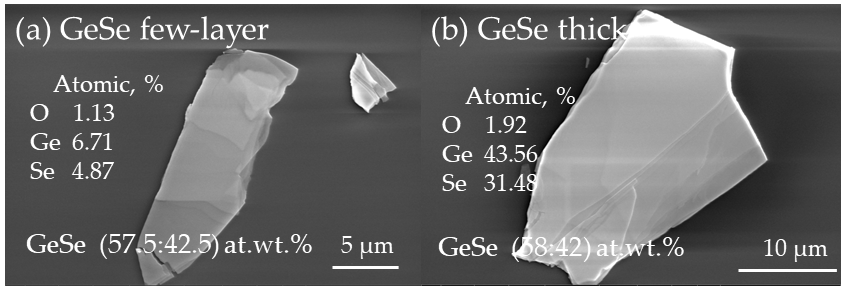

Supplement: Supplementary file 15 — Supplementary Figure 11. [file 41598_2023_46092_MOESM15_ESM.tif]

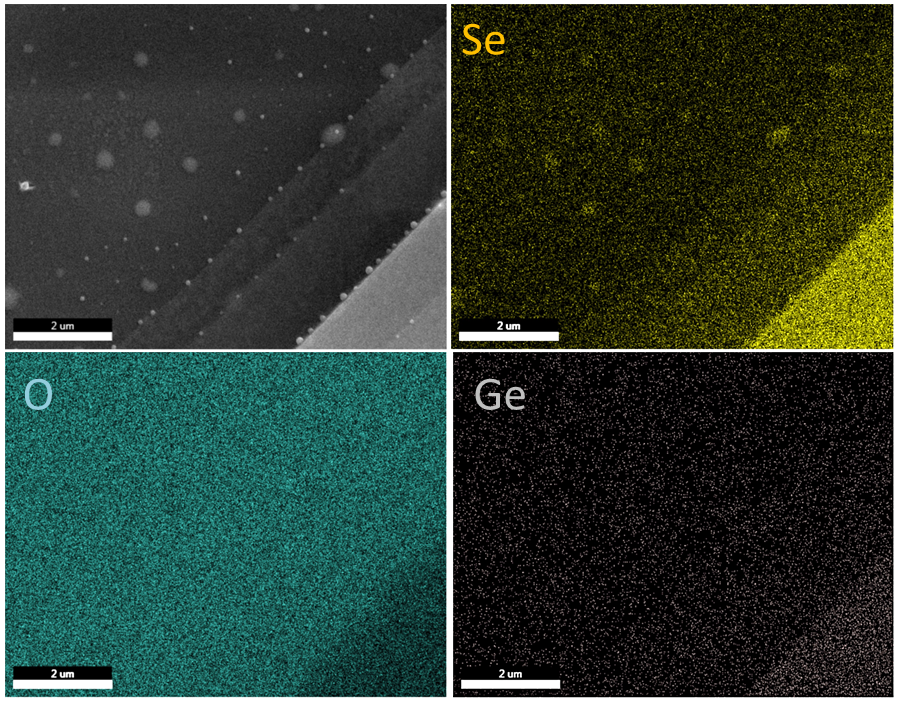

Supplement: Supplementary file 17 — Supplementary Figure 13. [file 41598_2023_46092_MOESM17_ESM.tif]

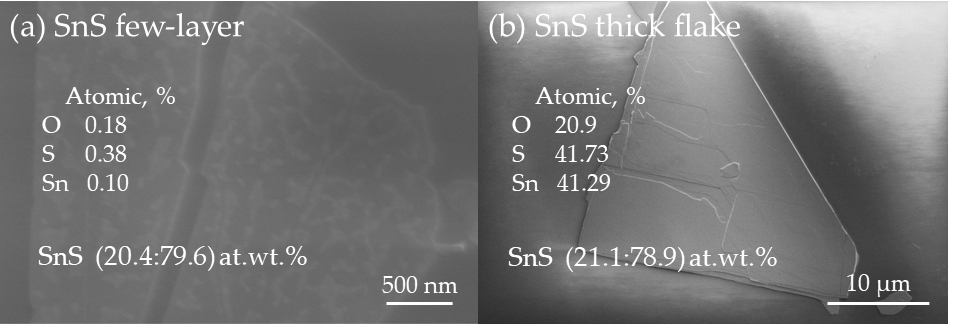

Supplement: Supplementary file 18 — Supplementary Figure 14. [file 41598_2023_46092_MOESM18_ESM.tif]

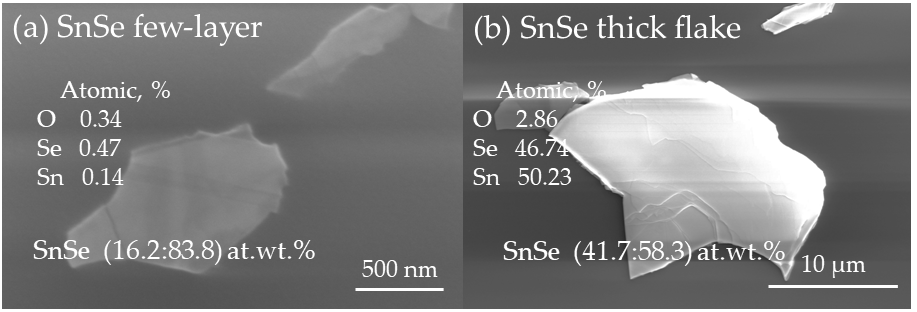

Supplement: Supplementary file 20 — Supplementary Figure 16. [file 41598_2023_46092_MOESM20_ESM.tif]

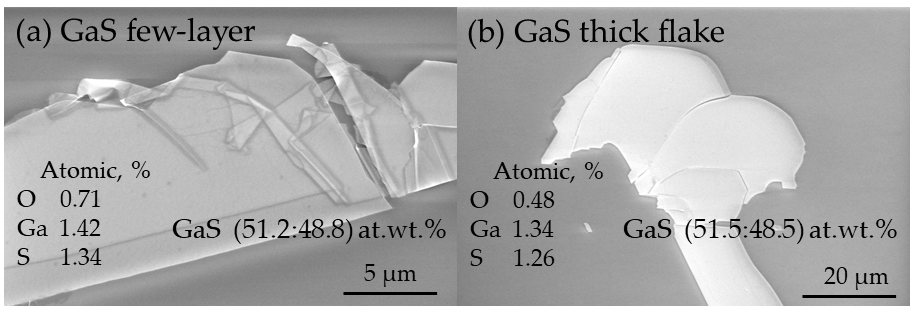

Supplement: Supplementary file 22 — Supplementary Figure 18. [file 41598_2023_46092_MOESM22_ESM.tif]

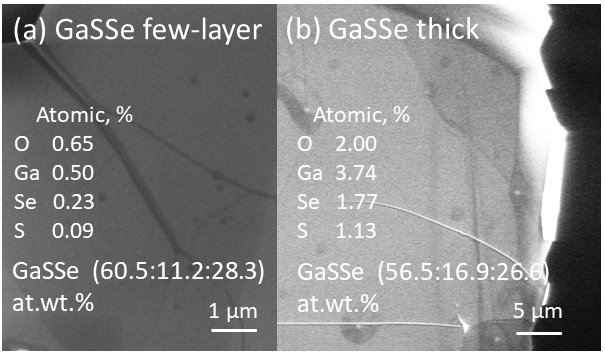

Supplement: Supplementary file 24 — Supplementary Figure 20. [file 41598_2023_46092_MOESM24_ESM.tif]
